# Mandatory large-scale food fortification programmes can reduce the estimated prevalence of inadequate zinc intake by up to 50% globally

**DOI:** 10.1038/s43016-024-00997-w

**Published:** 2024-06-19

**Authors:** K. Ryan Wessells, Mari S. Manger, Becky L. Tsang, Kenneth H. Brown, Christine M. McDonald

**Affiliations:** 1 Department of Nutrition and Institute for Global Nutrition, University of California, Davis, Davis, CA USA; 2 International Zinc Nutrition Consultative Group, San Francisco, CA USA; 3 Department of Pediatrics, School of Medicine, University of California, San Francisco, San Francisco, CA USA; 4 Food Fortification Initiative, Atlanta, GA USA

**Keywords:** Malnutrition, Developing world

## Abstract

Large-scale food fortification (LSFF) can increase dietary micronutrient intake and improve micronutrient status. Here we used food balance sheet data from the Food and Agriculture Organization of the United Nations to estimate current country-specific prevalences of inadequate zinc intake. We assessed the potential effects of improving existing LSFF programmes for cereal grains or implementing new programmes in 40 countries where zinc deficiency is a potential public health problem. Accounting for LSFF programmes as currently implemented, 15% of the global population (1.13 billion individuals) is estimated to have inadequate zinc intake. In countries where zinc deficiency is a potential public health problem, the implementation of high-quality mandatory LSFF programmes that include zinc as a fortificant would substantially increase the availability of zinc in the national food supply, reducing the estimated prevalence of inadequate zinc intake by up to 50% globally. Investments in strong LSFF programmes could have a substantial impact on population zinc status.

## Abstract (web summary)

Using food balance sheet data from the Food and Agriculture Organization of the United Nations, this study shows that mandatory large-scale food fortification programmes in the 40 countries where zinc deficiency is considered a public health problem could considerably reduce the prevalence of inadequate dietary zinc intake globally.

## Main

Many populations in low- and middle-income countries (LMICs) are vulnerable to zinc deficiency^1,2^. Among the 26 LMICs with nationally representative data available, 22 reported a prevalence of low plasma/serum zinc concentration (PZC) of >20%, indicative of a public health problem, in at least one physiological group^3–5^. Large-scale food fortification (LSFF), that is, post-harvest addition of essential micronutrients to an industrially processed food, can improve zinc intake and status in populations in LMICs. A recent meta-analysis found that zinc fortification significantly reduced the prevalence of zinc deficiency by 24–55% (ref. ^6^). LSFF is cost-effective, safe and deliverable through existing food systems without the need for changes in dietary intake patterns or behaviour^7–11^. As of August 2022, 82 LMICs had mandatory LSFF standards in place for at least one nutrient in at least one cereal grain (that is, wheat flour, maize flour or rice); however, zinc was a mandated nutrient in only 33 of these countries^12^.

Zinc fortification could clearly be a public health benefit in more LMICs. In 2021, the International Zinc Nutrition Consultative Group (IZiNCG) issued a call to action in which they identified 35 countries where zinc deficiency is a public health problem^13^ because (1) the percentage of the population at risk of inadequate zinc intake due to insufficient zinc in the national food supply was >25% and the prevalence of stunting among children less than 5 years of age was >20% or (2) the percentage of pre-school children or women of reproductive age with low PZC was ≥20% according to available national surveys^2,14^. Among these countries, 14 had mandatory LSFF standards for at least one cereal grain that included zinc fortificants, although in many cases compliance with the standard was suboptimal^12^. An additional 11 countries had mandatory LSFF standards that did not include zinc fortification and 10 had no mandatory LSFF standards for cereal grains. In countries where zinc deficiency is a public health problem, improving the performance of existing LSFF programmes (either by increasing programmatic compliance to existing standards or by expanding fortification standards to include zinc, or both) and establishing LSFF programmes in countries where such programmes do not exist could be a relatively low-cost, high-impact intervention^13^.

Thus, the objectives of this research were threefold. The first objective was to estimate country-specific prevalences of inadequate zinc intake based on food balance sheet data from the Food and Agriculture Organization of the United Nations (FAO) to update the list of countries where zinc deficiency is considered a public health problem (that is, the ‘baseline’); these analyses did not account for any LSFF programmes that may currently be in place. The second objective was to estimate country-specific prevalences accounting for LSFF programmes as currently implemented (that is, ‘current programme’) based on available data. The third objective was to estimate the potential effects of improving existing large-scale zinc fortification programmes for cereal grains or establishing new ones on the estimated prevalence of inadequate zinc intake in countries where zinc deficiency was considered a public health problem. To accomplish this third objective, we modelled three scenarios (Table 1): (1) achieving full industry compliance while retaining existing zinc fortification standards (that is, ‘full compliance’), (2) adding zinc to existing cereal grain standards (if not already included) or aligning existing standards that already include zinc fortification to reflect current international guidelines for zinc fortification with no changes to industry compliance (that is, ‘aligned standards’) and (3) establishing new standards, adding zinc to existing standards and/or aligning existing standards to reflect current international guidelines for zinc fortification for wheat flour, maize flour and rice while achieving full industry compliance (that is, ‘new/aligned standards with full compliance’).

**Table 1:** Overview of scenarios modelled to estimate the prevalence of inadequate zinc intake and potential impacts of improving food fortification programmes

Scenario	Zinc standard	Compliance	Countries to which the scenario was applied	Notes
Baseline	None	None	All countries	Used only to identify countries where zinc deficiency is a public health problem, assuming no LSFF programmes (that is, current LSFF programmes were not taken into account in this scenario)
Current programme	As reported in the GFDx	As reported in the GFDx (estimated if data were missing)	All countries	Accounts for LSFF programmes as currently implemented; estimates of inadequate dietary intake differ from the baseline only for countries with zinc fortification standards for wheat flour, maize flour and/or rice (zinc > 0 mg kg^−1^), industrial processing of >0% and industry compliance of >0%
Full compliance	As reported in the GFDx	100%	Countries with already established LSFF programmes where zinc deficiency is considered a public health problem	Estimates of inadequate dietary intake differ from the current LSFF programme only for countries with mandatory zinc fortification standards for wheat flour, maize flour and/or rice (zinc > 0 mg kg^−1^) and industrial processing of >0%; full compliance was assumed for all cereal grains with zinc fortification standards
Aligned standards	In countries with any mandatory LSFF standard for wheat flour, maize flour or rice; zinc added to existing standard or standard aligned to reflect current zinc fortification guidelines	As reported in the GFDx (estimated if data were missing)	Countries with already established LSFF programmes where zinc deficiency is considered a public health problem	Estimates of inadequate dietary zinc intake differ from the current LSFF programme for all countries with current wheat flour, maize flour or rice food fortification programmes (zinc ≥ 0 mg kg^−1^), that is, whether or not zinc is included as a fortificant in the current programme, industrial processing of >0% and industry compliance of >0%
New/aligned standards with full compliance	Assumes mandatory LSFF standards for wheat flour, maize flour or rice (or all three combined); zinc added to existing standard or new standard/standard aligned to reflect current zinc fortification guidelines	100%	All countries where zinc deficiency is considered a public health problem	Estimates of inadequate dietary zinc intake differ from the current LSFF programme for all countries with industrial processing of >0% for wheat flour, maize flour and/or rice

For all scenarios, the data are based on the 2018 FAO food balance sheet data, a composite nutrient composition database, IZiNCG physiological requirements, the Miller equation to estimate zinc absorption and an assumed 25% inter-individual variation in zinc intake. The ‘baseline’ scenario does not account for current LSFF programmes. The ‘current programme’ scenario reflects fortification as currently implemented in all countries with current mandatory or voluntary fortification standards (whether or not zinc fortification is included in the standards) for wheat flour, maize flour and/or rice (current zinc standard and current percentage compliance; *n* = 87). The ‘full compliance’ scenario reflects retaining current standards (whether or not zinc fortification is included in the standards), but where compliance with mandatory fortification is increased to 100%. This scenario applied only to countries where zinc deficiency is considered a public health problem and with current mandatory fortification standards (*n* = 29). The ‘aligned standards’ scenario reflects either adding zinc to the mandatory standard (if it is not already included) or aligning the standard to reflect current zinc fortification guidelines. This scenario applied only to countries where zinc deficiency is considered a public health problem and with current mandatory fortification standards (*n* = 29). This ‘new/aligned standards with full compliance’ scenario reflects mandatory zinc fortification standards, aligned with current guidelines, for each staple food independently and combined with 100% compliance with the standards. This scenario only applied to countries where zinc deficiency is considered a public health problem (*n* = 40). Although not a primary objective of this analysis, estimates from each hypothetical scenario detailed above were also generated for all countries with available data to provide information to relevant stakeholders (Supplementary Table 3).

## Results

### Countries where zinc is a public health problem

Globally, without accounting for any current LSFF programmes (the ‘baseline’ scenario), 16.4% of the world’s population is estimated to have inadequate zinc intake based on national food balance sheet data. In these updated analyses, zinc deficiency was identified as a public health problem in 40 countries: 18 countries had an estimated prevalence of inadequate zinc intake of >25% and a prevalence of stunting among children under 5 years of age of >20%, 18 countries had a prevalence of low PZC among women of reproductive age and/or pre-school children of >20%, and 4 countries had elevated prevalences of all three criteria (Table 2 and Supplementary Table 1). These countries are largely situated in South and Southeast Asia and sub-Saharan Africa.

**Table 2:** Countries where zinc deficiency is considered a public health problem

Country	Criteria for zinc deficiency as a public health problem	Estimated prevalence of inadequate zinc intake (baseline) (%)	Prevalence of stunting in children (%)	Year of stunting data	Prevalence of low PZC in children (%)	Prevalence of low PZC in women (%)	Year of PZC data
South Asia							
Afghanistan	PZC	20.9	38.2	2018	15.1	**23.4**	2013
Bangladesh	PZC	24.8	28	2019	**44.6**	**57.3**	2012
India	FBS (stunting), PZC	**29.2**	**34.7**	2017	18.9	**28.4**	2018
Nepal	PZC	20.2	31.5	2019	**20.7**	**24.3**	2016
Pakistan	PZC	19.7	37.6	2018	18.6	**22.1**	2018
Sub-Saharan Africa							
Botswana	FBS (stunting)	**27.4**	**28.9**	2007			
Burkina Faso	FBS (stunting)	**35.2**	**24.9**	2020			
Burundi	FBS (stunting)	**38.3**	**50.9**	2020			
Cameroon	FBS (stunting), PZC	**26.0**	**28.9**	2018	**82.6**	**81.6**	2009
Chad	FBS (stunting)	**33.1**	**31.1**	2021			
Comoros	FBS (stunting)	**28.2**	**31.1**	2012			
Côte d’Ivoire	FBS (stunting)	**28.3**	**21.6**	2016			
Democratic Republic of the Congo	FBS (stunting)	**38.8**	**41.8**	2017			
Eswatini	FBS (stunting)	**25.2**	**25.5**	2014			
Ethiopia	PZC	10.2	36.8	2019	**35**	**33.8**	2015
Kenya	FBS (stunting), PZC	**25.0**	**26.2**	2014	**81.6**	**79.9**	2011
Lesotho	FBS (stunting)	**34.2**	**34.6**	2018			
Malawi	FBS (stunting), PZC	**26.9**	**34.9**	2020	**60.4**	**62.5**	2016
Mozambique	FBS (stunting)	**28.5**	**37.5**	2020			
Nigeria	PZC	17.7	31.5	2020	**20**	**43.8**	2001
Rwanda	FBS (stunting)	**28.9**	**33.1**	2020			
Senegal	PZC	24.4	17.9	2019	**50**	**59**	2010
South Africa	PZC	15.8	21.4	2017	**51**		2005
United Republic of Tanzania	FBS (stunting)	**26.3**	**31.8**	2018			
Zambia	FBS (stunting)	**36.9**	**34.6**	2018			
Zimbabwe	FBS (stunting)	**45.4**	**23.5**	2019			
Central Asia, North Africa and Middle East							
Islamic Republic of Iran	PZC	23.3	4.8	2017	19.1	**28**	2015
Yemen	FBS (stunting)	**25.9**	**46.4**	2013			
Central and Andean Latin America and Caribbean							
Colombia	PZC	9.3	12.7	2016	**43**		2010
Costa Rica	PZC	14.0	9	2018	**23.9**		2009
Ecuador	PZC	11.5	23	2019	**28**	**56**	2012
Guatemala	FBS (stunting)	**28.5**	**46.7**	2015	13.3	18.3	2016
Haiti	FBS (stunting)	**29.1**	**21.9**	2017			
Mexico	PZC	14.9	13.9	2020	**26.6**	**33.8**	2006
East and Southeast Asia and Pacific							
Cambodia	PZC	19.2	32.4	2014	**67.5**	**62.8**	2014
Indonesia	FBS (stunting)	**26.4**	**30.8**	2018			
Maldives	PZC	25.9	15.3	2017	16	**27**	2007
Philippines	PZC	11.2	28.8	2019	17.9	**28.4**	2013
Timor-Leste	PZC	21.5	46.7	2020	**60.3**		2013
Vietnam	PZC	8.0	19.6	2020	**51.9**	**67.2**	2010

Country-specific data that meet these criteria are presented in bold font. FBS, food balance sheets.

### Current status of LSFF programmes

Twenty-nine of the 40 countries where zinc deficiency is considered a public health problem currently have legislation mandating micronutrient fortification of one or more cereal grains (that is, wheat flour, maize flour and/or rice). However, only 17 of them have one or more standards that include a zinc fortificant (Table 3 and Supplementary Table 2). For those with mandatory zinc fortification standards, zinc levels range widely among countries (7.5–101.3 mg kg^−1^), with the majority being below international guidelines^15–17^, based on the assumed milling extraction rate and estimated per capita consumption of the cereal grain. The median (1st and 3rd quartiles) percentage of cereal grains that are industrially processed in these 17 countries are 100% (71.5%, 100%) for wheat flour and 35% (7.5%, 42.5%) for maize flour; the estimated median compliance with the fortification standard also varied widely, with 71.2% (20.0%, 86.0%) compliance for wheat flour and 40.0% (5.5%, 77.5%) for maize flour.

**Table 3:** Current fortification standards in countries where zinc deficiency is considered a public health problem

Country	Wheat flour			Maize flour			Rice		
	Zinc standard (mg kg^−1^)	Industrially processed (%)	Fortification compliance (%)	Zinc standard (mg kg^−1^)	Industrially processed (%)	Fortification compliance (%)	Zinc standard (mg kg^−1^)	Industrially processed (%)	Fortification compliance (%)
**South Asia**									
Afghanistan	50 (M)	63	71.2	NA	0	NA	NA	55	NA
Bangladesh	27 (V)	78	–	NA	0	NA	40 (V)	28	1
India	12.5 (V)	32	–	NA	0	NA	12.5 (V)	50	–
Nepal	0 (M)	25	36.8	NA	99	NA	NA	27	NA
Pakistan	NA	32	NA	NA	0	NA	NA	90	NA
**Sub-Saharan Africa**									
Botswana	NA	100	NA	NA	98	NA	NA	100	NA
Burkina Faso	0 (M)	100	61.5	NA	0	NA	NA	72	NA
Burundi	88 (M)	40	–	49 (M)	0	NA	NA	30	NA
Cameroon	95 (M)	100	100	NA	100	NA	NA	78	NA
Chad	0 (M)	38	0	0 (M)	0	0	NA	7	NA
Comoros	NA	100	NA	NA	0	NA	NA	100	NA
Côte d’Ivoire	0 (M)	100	99	NA	100	NA	NA	51.12	NA
Democratic Republic of the Congo	NA	100	NA	NA	5	NA	NA	5	NA
Eswatini	20 (V)	100	–	NA	0	NA	NA	100	NA
Ethiopia	80 (M)	39	0	NA	30	NA	NA	0	NA
Kenya	40 (M)	100	100	30 (M)	51	0	NA	83	NA
Lesotho	0 (M)	100	66	0 (M)	71	0	NA	100	NA
Malawi	80 (M)	100	20	40 (M)	15	40	NA	6	NA
Mozambique	30 (M)	100	60	20 (M)	30	70	NA	68	NA
Nigeria	50 (M)	96	74	50 (M)	0	80	NA	80	NA
Rwanda	60 (M)	60	10	49 (M)	35	40	NA	40	NA
Senegal	0 (M)	72	96	NA	0	NA	NA	100	NA
South Africa	15 (M)	100	80	15 (M)	75	83.4	NA	100	NA
United Republic of Tanzania	40 (M)	99.5	86	22.5 (M)	45	7	NA	0	NA
Zambia	0 (V)	100	–	NA	35	NA	NA	37	NA
Zimbabwe	40 (M)	80	40	40 (M)	38	5	NA	100	NA
**Central Asia, North Africa and Middle East**									
Islamic Republic of Iran	0 (M)	100	–	NA	0	NA	NA	60	NA
Yemen	0 (M)	100	–	NA	0	NA	NA	100	NA
**Central and Andean Latin America and Caribbean**									
Colombia	0 (M)	100	81.3	NA	0	NA	NA	80	NA
Costa Rica	0 (M)	100	100	0 (M)	100	100	7.5 (M)	100	100
Ecuador	0 (M)	100	–	NA	0	NA	NA	0	NA
Guatemala	0 (M)	100	100	15 (M)	0	100	NA	94	NA
Haiti	0 (M)	100	75	NA	10	NA	NA	90	NA
Mexico	40 (M)	100	0	40 (M)	40	3.1	NA	100	NA
**East and Southeast Asia and Pacific**									
Cambodia	NA	100	NA	NA	3	NA	NA	0	NA
Indonesia	30 (M)	100	87	NA	0	NA	NA	7	NA
Maldives	NA	100	NA	NA	0	NA	NA	100	NA
Philippines	0 (M)	100	–	NA	0	NA	0 (M)	29	–
Timor-Leste	NA	100	NA	NA	0	NA	NA	100	NA
Vietnam	101.3 (M)	100	–	NA	0	NA	NA	20	NA

The GFDx^12^ was used to obtain information on the presence or absence of a mandatory or voluntary food fortification programme in each country and which staple foods were fortified under the aforementioned programme (that is, wheat flour, maize flour and/or rice; GFDx Indicators 1 and 9), whether zinc was included as a fortificant in the programme (standard in mg kg^−1^; GFDx Indicator 6), the percentage of each staple food that was industrially processed (GFDx Indicator 12) and the current percentage compliance with fortification standards (GFDx Indicator 15). The data are as reported in the GFDx and are the latest available data (regardless of year). The data on compliance with fortification were not available for all countries. If the data were unavailable (indicated by ‘−’), the estimated compliance with mandatory fortification was calculated for all modelled scenarios as the median compliance with fortification for all other countries on the same continent with mandatory fortification standards for each food commodity; compliance with voluntary fortification was assumed to be zero. M, mandatory fortification; NA, not applicable (no or unknown fortification); V, voluntary fortification.

### Inadequate zinc intake based on current LSFF programmes

Accounting for LSFF programmes as currently implemented (that is, existing standards and actual reported/estimated compliance, the ‘current programme’ scenario), 15% of the global population (1.13 billion individuals) is estimated to have inadequate zinc intake (Table 4). The regional estimated prevalence of inadequate zinc intake ranges from 6% in southern and tropical Latin America to 27% in South Asia. Of the 1.13 billion people globally who are estimated to have inadequate zinc intake currently, 43% reside in South Asia.

**Table 4:** Regional means of national data on the estimated prevalence of inadequate zinc intake in the population in relation to simulated modifications of LSFF programmes in countries where zinc deficiency is considered a public health problem

Region	Number of countries	Number of countries with mandatory LSFF of cereal grains	Population (million)	Baseline (%)	Current programme (%)	Full compliance (%)	Aligned standards (%)	New/aligned standards with full compliance, wheat flour only (%)	New/aligned standards with full compliance, maize flour only (%)	New/aligned standards with full compliance, rice only (%)	New/aligned standards with full compliance, combined (%)
South Asia	5	2	1,791.5	27.4 ± 3.5	27.0 ± 4.7	27.0 ± 4.8	26.9 ± 4.9	11.4 ± 2.6	26.8 ± 5.4	9.2 ± 1.4	5.1 ± 0.9
Sub-Saharan Africa	45	29	1,048.5	22.2 ± 8.7	16.6 ± 10.1	14.5 ± 10.7	13.6 ± 10.5	10.6 ± 6.9	12.3 ± 9.7	12.5 ± 10	7.6 ± 7.3
Central Asia, North Africa and Middle East	25	11	604.2	16.4 ± 6.7	16.2 ± 6.9	16.2 ± 6.9	12.4 ± 7.1	12.2 ± 7.4	16.2 ± 6.9	14.0 ± 5.9	12.2 ± 7.5
Central and Andean Latin America and Caribbean	26	26	354.4	16.5 ± 6.6	16.1 ± 6.5	12.9 ± 8.5	13.1 ± 7.1	10.0 ± 7.8	12.3 ± 8.6	12.6 ± 7.2	8.3 ± 8.8
East and Southeast Asia and Pacific	20	8	707.1	19.2 ± 9.0	14.2 ± 7.5	13.7 ± 7.6	11.0 ± 8.7	10.5 ± 8.6	14.2 ± 7.4	11.4 ± 8.5	9.5 ± 9.1
Central and Eastern Europe	20	1	323.8	8.0 ± 2.4	8.0 ± 2.4	8.0 ± 2.4	8.0 ± 2.4	8.0 ± 2.4	8.0 ± 2.4	8.0 ± 2.4	8.0 ± 2.4
China	1	0	1,427.6	6.6 ± 0	6.6 ± 0	6.6 ± 0	6.6 ± 0	6.6 ± 0	6.6 ± 0	6.6 ± 0	6.6 ± 0
Southern and tropical Latin America	5	5	283	5.7 ± 2.2	5.7 ± 2.2	5.7 ± 2.2	5.7 ± 2.2	5.7 ± 2.2	5.7 ± 2.2	5.7 ± 2.2	5.7 ± 2.2
High-income countries	27	5	1,003.9	8.1 ± 3.9	8.1 ± 3.9	8.1 ± 3.9	8.1 ± 3.9	8.1 ± 3.9	8.1 ± 3.9	8.1 ± 3.9	8.1 ± 3.9
Global	174	87	7,544.6	16.4 ± 9.9	15.0 ± 9.6	14.5 ± 9.8	13.8 ± 9.8	9.5 ± 5.4	14.2 ± 9.7	9.6 ± 5.9	7.4 ± 5.5
Global population estimated to have inadequate zinc intake (million)				1,237.3	1,132.0	1,094.3	1,042.6	716.7	1,068.8	723.9	560.2

Estimates were calculated using the composite nutrient composition database, IZiNCG physiological requirements, the Miller equation to estimate zinc absorption and an assumed 25% inter-individual variation in zinc intake. The regional data are presented first for LMICs in descending order according to the estimated prevalence of inadequate zinc intake and then for high-income countries. The data represent 174 countries and are presented as the mean ± s.d. and are weighted by national population size.

Seventeen of the 40 countries where zinc is considered a public health problem have an existing mandatory zinc fortification programme; in 6 of these countries, the fortification programme (as currently implemented) was responsible for an estimated reduction in the prevalence of inadequate zinc intake of more than 10 percentage points compared with the ‘baseline’ scenario (Table 5). The estimated prevalence of inadequate zinc intake remained at >25% in only 4 of these 17 countries. In comparison, the estimated prevalence of inadequate zinc intake was >25% in 13 of the 23 countries without mandatory zinc fortification standards. However, even with mandatory LSFF programmes as currently implemented, around 736 million individuals are estimated to have inadequate zinc intake in countries where zinc deficiency is considered a public health problem (Table 4).

**Table 5:** Country-specific estimated prevalences of inadequate zinc intake in the population in countries where zinc deficiency is considered a public health problem

Country	Current programme (%)	Full compliance (%)	Aligned standards (%)	New/aligned standards with full compliance, wheat flour only (%)	New/aligned standards with full compliance, maize flour only (%)	New/aligned standards with full compliance, rice only (%)	New/aligned standards with full compliance, combined (%)
**South Asia**							
Afghanistan^a^	4.9	3.3	3.4	2.4	4.8	3.9	2.1
Bangladesh	24.4	24.4	24.4	9.3	24.4	5.5	3.4
India	29.2	29.2	29.2	12.7	29.2	9.6	5.7
Nepal	20.2	20.2	16.2	11.6	4.7	9.7	2.7
Pakistan	19.7	19.7	19.7	6.0	19.7	10.1	4.1
**Sub-Saharan Africa**							
Botswana	27.4	27.4	27.4	4.8	3.4	12.5	1.5
Burkina Faso	35.2	35.2	22.8	17.7	35.2	14.2	8.4
Burundi^a^	30.9	29.1	30.4	28.4	30.9	25.8	23.8
Cameroon^a^	7.4	7.4	7.3	7.3	2.4	3.6	1.7
Chad	33.1	33.1	33.1	29.6	33.1	31.5	28.1
Comoros	28.2	28.2	28.2	5.3	28.2	1.8	1.2
Côte d’Ivoire	28.3	28.3	6.3	6.3	4.8	6.2	1.4
Democratic Republic of the Congo	38.8	38.8	38.8	20.0	33.2	35.4	16.1
Eswatini	25.2	25.2	25.2	5.0	25.2	5.6	2.3
Ethiopia^a^	10.2	5.4	10.2	6.3	4.9	10.2	3.5
Kenya^a^	9.3	5.3	6.4	6.4	3.2	4.9	1.9
Lesotho	34.2	34.2	12.5	8.4	3.6	24.3	2.0
Malawi^a^	20.5	11.9	17.9	14.2	11.9	20.1	8.7
Mozambique^a^	15.4	11.1	5.0	4.1	7.7	4.0	1.5
Nigeria^a^	9.1	7.5	5.7	4.3	9.1	2.9	1.9
Rwanda^a^	25.8	17.2	23.5	15.8	19.3	19.1	9.7
Senegal	24.4	24.4	7.9	7.7	24.4	2.0	1.4
South Africa^a^	7.2	6.3	2.0	3.1	2.5	4.2	1.4
United Republic of Tanzania^a^	16.7	10.7	9.4	8.9	4.8	16.7	3.4
Zambia	36.9	36.9	36.9	20.8	6.4	33.9	4.5
Zimbabwe^a^	33.1	8.7	22.7	11.1	6.6	10.9	2.3
**Central Asia, North Africa and Middle East**							
Islamic Republic of Iran	23.3	23.3	3.8	2.6	23.3	13.3	2.3
Yemen	25.9	25.9	2.5	1.7	25.9	9.1	1.3
**Central and Andean Latin America and Caribbean**							
Colombia	9.3	9.3	4.3	3.7	9.3	2.8	1.8
Costa Rica^a^	11.1	11.1	1.6	4.4	5.2	2.9	1.6
Ecuador	11.5	11.5	2.2	2.2	11.5	11.5	2.2
Guatemala^a^	28.5	28.5	9.2	9.2	28.5	19.9	7.3
Haiti	29.1	29.1	6.8	4.9	23.0	3.1	1.6
Mexico^a^	14.5	5.3	14.2	5.7	4.4	11.0	2.5
**East and Southeast Asia and Pacific**							
Cambodia	19.2	19.2	19.2	15.2	18.7	19.2	14.8
Indonesia^a^	14.1	13.0	9.2	8.1	14.1	10.3	6.4
Maldives	25.9	25.9	25.9	4.1	25.9	4.7	2.1
Philippines	11.2	11.2	2.3	2.9	11.2	3.9	1.8
Timor-Leste	21.5	21.5	21.5	4.9	21.5	2.3	1.4
Vietnam^a^	5.7	5.0	5.8	5.1	5.7	3.7	3.5
**All countries where zinc deficiency is considered a public health problem**							
Population estimated to have inadequate zinc intake (millions)	735.8	698.1	646.4	320.5	672.6	327.7	164.0

Estimates were calculated using the composite nutrient composition database, IZiNCG physiological requirements, the Miller equation to estimate zinc absorption and an assumed 25% inter-individual variation in zinc intake. Although not a primary objective of this analysis, estimates from each hypothetical scenario are presented for all countries in Supplementary Table 3, to make information available to relevant stakeholders.

^a^Countries with a current mandatory fortification programme for wheat flour, maize flour and/or rice that includes zinc as a fortificant.

### Inadequate zinc intake based on modelled LSFF programmes

The median (1st and 3rd quartiles) estimated industry compliance with existing national fortification standards was 65% (9.3%, 84%) across all mandatory LSFF programmes in the 17 countries where zinc deficiency is considered a public health problem and mandatory zinc fortification standards for at least one cereal grain are in place. Increasing compliance with fortification to 100% in these 17 countries while maintaining current zinc standards (that is, the ‘full compliance’ scenario) led to an estimated relative reduction of >25% in the prevalence of inadequate dietary zinc intake (compared with the ‘current programme’ scenario) in 9 countries, while only 2 countries still had estimated prevalences of inadequate zinc intake >25% (that is, Burundi and Guatemala; Table 5). However, across the 40 countries where zinc deficiency is considered a public health problem, around 698 million individuals would continue to have inadequate zinc intake (Table 5) as there would be only a 0.5 percentage point reduction in the estimated global prevalence of inadequate zinc intake, from 15% to 14.5% (Table 4).

Among the countries where zinc deficiency was identified as a public health problem, 29 countries already have mandatory LSFF of at least one cereal grain in place. In these countries, there is an immediate opportunity to expand current mandatory fortification standards to include zinc (12 countries) or to align zinc standards with current international guidelines (17 countries; Table 3 and Extended Data Table 1), even without changing compliance with the standards. In the ‘aligned standards’ scenario, the estimated relative reduction in the prevalence of inadequate zinc intake (compared with the ‘current programme’ scenario) was >25% in 20 of the 29 countries, while only 2 countries with existing LSFF programmes continued to have estimated prevalences of inadequate zinc intake >25% (that is, Burundi and Chad; Table 5). Yet, around 646 million individuals in countries where zinc deficiency is considered a public health problem would continue to have inadequate zinc intake and the estimated global prevalence of inadequate zinc intake would remain close to the ‘current programme’ scenario at 13.8% (Table 5).

**Fig. 1:**
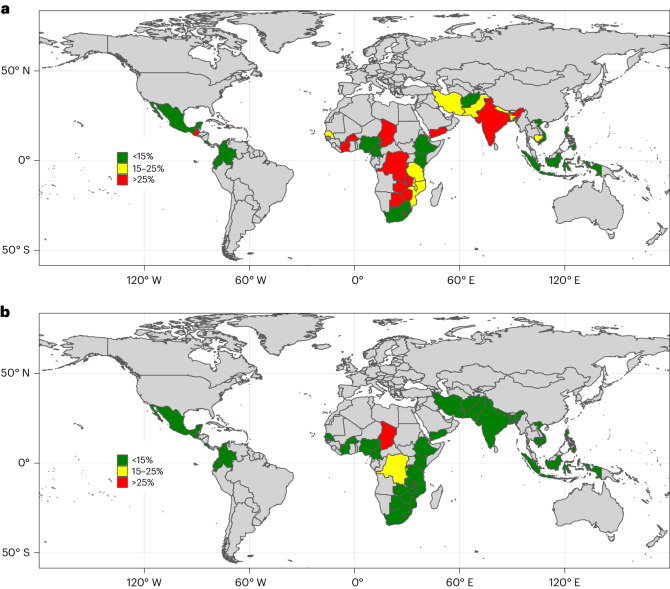
Estimated national prevalences of inadequate zinc intake for countries where zinc deficiency is considered a public health problem. **a**,**b**, Estimated prevalences of inadequate zinc intake considering LSFF programmes as currently implemented (existing standards and actual reported or estimated compliance) (**a**) and under the ‘new/aligned standards with full compliance’ scenario (**b**) (*n* = 40). Zinc deficiency was considered a public health problem in countries where (1) the percentage of the population at risk of inadequate zinc intake due to inadequate zinc in the national food supply was >25% and the prevalence of stunting among children less than 5 years of age was >20%, or (2) the percentage of pre-school children or women of reproductive age with low PZC was >20% according to available national surveys^2^^,^^14^. The estimates of inadequate zinc intake in the national food supply were calculated using the composite nutrient composition database, IZiNCG physiological requirements, the Miller equation to estimate zinc absorption^36^ and an assumed 25% inter-individual variation in zinc intake^22^. Source data

If the 40 countries where zinc deficiency was identified as a public health problem were to implement LSFF programmes with wheat flour, maize flour and rice fortified with zinc levels consistent with international guidelines (Extended Data Table 1) and with 100% compliance of all industrially processed staple foods (that is, the ‘new/aligned standards with full compliance’ scenario), the prevalence of inadequate intake in these countries would decrease by 78%, from 736 million to 164 million (Table 5). Only one country, Chad, would have an estimated prevalence of inadequate zinc intake >25% (Table 5 and Fig. 1b). The estimated global prevalence of inadequate zinc intake would decrease by approximately 50%, from 15.0% to 7.4% (Table 4). Even assuming suboptimal compliance (that is, 85%) with the ‘new/aligned standards’, the prevalence of inadequate zinc intake would decrease markedly to 7.8%.

If each country where zinc deficiency is considered a public health problem were to implement the LSFF of only one cereal grain (for example, wheat flour, maize flour or rice), with zinc standards reflective of current international fortification guidelines and full compliance, only two countries where zinc deficiency is considered a public health problem would still have an estimated risk of inadequate zinc intake >25%. The most effective vehicle for the greatest reduction in zinc deficiency would be wheat flour for 14 countries, maize flour for 13 countries and rice for 13 countries (Table 5).

## Discussion

The results of our analyses suggest that the zinc content of national food supplies may be inadequate to meet zinc requirements for approximately 15% of the world’s population, with country-specific estimates ranging from 2% to 39%. Despite recent efforts to scale-up LSFF programmes in LMICs, the estimated global risk of inadequate zinc intake has not changed markedly in the past 10–15 years^18^. Using a novel approach to analyse national food balance sheet data, we have demonstrated the potential for zinc fortification to have a substantial impact on estimated dietary zinc intake in countries where zinc deficiency is a public health problem if investments are made to introduce mandatory LSFF programmes, expand LSFF programmes to include zinc, align zinc standards with current international guidelines and improve industry compliance. The implementation of high-quality mandatory LSFF of cereal grains (that is, wheat flour, maize flour and rice) in the 40 countries where zinc deficiency is considered a public health problem could increase the availability of zinc in the national food supply, thus reducing the estimated global prevalence of inadequate zinc intake by up to 50%. However, fortification opportunities differ widely among countries due to differences in the percentage of the grains industrially fortified and the daily per capita availability of the fortification vehicles, resulting in country-specific estimated relative reductions ranging from 15% to 96%.

Although LSFF programmes are considered cost-effective and safe and are deliverable through existing food systems without requiring changes in dietary intake patterns^9^, fewer than half of the countries where zinc deficiency is considered a public health problem currently have LSFF programmes that include zinc as a fortificant. Important barriers to zinc fortification include limited information on population zinc status, the absence of national policies related to the prevention of zinc deficiency and the exclusion of zinc from regional fortification standards^8^. Furthermore, to be effective, LSFF programmes must fortify appropriate food vehicle(s), add micronutrients at the appropriate concentrations and ensure compliance to standards. Among the countries that currently implement LSFF programmes that include zinc, the zinc standards are often below international guidelines and/or monitoring and quality assurance systems are inadequate^19,20^. Thus, although comprehensive high-quality LSFF programmes in all 40 countries where zinc deficiency is considered a public health problem would have the largest impact on zinc intake globally, even limited changes to current LSFF programmes have the potential for an immediate impact on the zinc intake of populations in countries with existing programmes. For the 17 countries with current zinc LSFF programmes, either implementing strong monitoring and evaluation to improve compliance in their current programmes (that is, ‘full compliance’) or aligning their current zinc fortification standards with international guidelines (‘aligned standards’) could lead to an ~30% relative reduction in the overall estimated prevalence of inadequate zinc intake. As expected, larger relative reductions were observed among countries with a favourable fortification opportunity (that is, a high percentage of the fortifiable food being industrially processed and adequate daily per capita availability of the fortified food) but with a current programme that is suboptimally designed or implemented (for example, low compliance with current standards and zinc standards below international guidelines). For the additional 12 countries that currently have mandatory LSFF programmes that do not include zinc, overcoming barriers to zinc fortification and adding zinc to the existing programme in accordance with current guidelines (‘aligned standards’) could lead to an ~65% relative reduction in the overall estimated prevalence of inadequate zinc intake, even without improving industry compliance.

Our analyses have several strengths. First, we used publicly available data (that is, food balance sheets) to obtain information on the adequacy of zinc in the national food supplies for the majority of LMICs as a proxy indicator of zinc deficiency in the absence of comprehensive biochemical data^1^. According to a recent study, one barrier to the design and implementation of zinc fortification is limited information on population zinc status^8^. Second, we employed a novel method, using country-level data available through the Global Fortification Data Exchange (GFDx; that is, information on fortification standards, percentage of cereal grains industrially processed and reported compliance with LSFF) and alternative model scenarios, to examine the potential impacts of LSFF with zinc on the adequacy of zinc in national food supplies. A previous analysis using similar methods reported that achieving crop breeding targets and universal uptake of zinc-biofortified maize and bean crops could have a similar impact, reducing the estimated risk of zinc deficiency in Africa by 43% (ref. ^21^). These analyses provide a ‘gold standard’ of what could be achieved in an ideal world and provide country-, regional- and global-level stakeholders with preliminary evidence that can be used as a starting point to further explore fortification opportunities in these countries.

However, the analyses also have several limitations that need to be recognized in the interpretation and use of the results^22^. First, methodological assumptions regarding the nutrient composition of foods, zinc requirements and zinc absorption may affect estimates of the prevalence of inadequate zinc intake from food balance sheets, and estimates published by different research groups tend to vary widely^18,21,23,24^. In addition, different types of data (for example, PZC, food balance sheets and stunting) tend to identify different countries as having zinc deficiency as a public health problem^25^. Finally, biochemical evidence suggests that the prevalence of zinc deficiency is substantially higher than food balance sheet data would suggest. Thus, food balance sheets probably underestimate the true extent of zinc deficiency. As nationally representative surveys with PZC data become available for more countries, we expect that zinc deficiency will be identified as a public health problem in more countries^4,25^.

An additional limitation of these analyses is that food balance sheets provide data on annual national food availability; they do not reflect actual dietary intake and do not account for inter- and intrahousehold differences in intake. However, a recent analysis of global dietary data indicated that micronutrient intake distributions vary widely not only by country but also by sex and age^26^. In addition, a recent analysis of national surveys indicated that zinc deficiency is associated with socio-economic status and place of residence (for example, urban versus rural)^3^. Furthermore, the models assume that the reach of the industrially processed cereal grains is 100% (that is, all individuals in the population are consuming the potentially fortifiable food) and that there are no inter- or intrahousehold disparities in the amount of the potentially fortifiable food vehicle consumed. However, evidence from LSFF programmes indicates that vulnerable or at-risk populations often do not have equitable access to, or benefit from, industrially processed cereal grains fortified by LSFF^27^. Thus, although the results of our analyses provide preliminary insights, additional information on the household consumption of fortifiable food vehicles is needed to determine whether higher-risk population subgroups would benefit from the LSFF programmes^27–29^. Finally, the estimates from the ‘new/aligned standards with full compliance’ model are based on the fortification of wheat flour, maize flour and rice in all countries and present an ‘ideal’ or ‘best possible’ scenario that may not be appropriate, feasible or cost-effective in all countries. In addition, this scenario could lead to an excess intake of zinc in some segments of the population, although it has been suggested that the current tolerable upper levels of intake for zinc should be reassessed^30^. In the design and implementation of actual LSFF programmes, countries need to not only consider scientific and technical factors but also ensure adequate governance and political commitment^20^. Programmes need to ensure a strong coordination mechanism, effective regulatory monitoring and technical capacity building among food processors to be successful^31–33^. Many current LSFF programmes are not achieving their potential impact due to suboptimal design and/or implementation^20,34^. However, if countries can leverage what they learn from current programmes to increase their effectiveness and address critical roadblocks, national mandatory LSFF programmes could potentially have a large impact on the prevalence of inadequate zinc intake, both nationally and globally. In addition, alternative zinc intervention strategies (for example, targeted multiple micronutrient supplementation, home fortification and biofortification) may need to be considered in some settings where the majority of cereal grains are locally produced and processed or where large segments of the population do not have access to industrially processed cereal grains and/or for particularly vulnerable subgroups (for example, infants and young children and pregnant women)^20,34^.

Despite these limitations, our results can be used to inform the modification or establishment of LSFF programmes. In all cases, country- and context-specific LSFF programmes are essential. For the 22 countries where zinc deficiency is identified as a public health problem based on PZCs measured in nationally representative surveys, zinc intervention strategies, such as LSFF, should be considered immediately if a favourable food vehicle opportunity exists. The other 18 countries where zinc deficiency has been identified as a public health problem based on estimated inadequate dietary zinc intakes and the prevalence of stunting among children under 5 years should consider incorporating the assessment of PZC in their upcoming national nutrition surveys^35^ while simultaneously exploring the potential to implement or improve national food fortification programmes.

These analyses indicate that the availability of zinc in the national food supply is insufficient in a sizable number of LMICs and that the corresponding estimated prevalence of inadequate zinc intake is high in many of them. Our findings illustrate the potential for reductions in the estimated prevalence of inadequate zinc intake in countries where zinc deficiency is a public health problem when a successful food fortification approach is employed. These findings can be used to preliminarily inform country- and context-specific LSFF programmes for cereal grains in individual countries. As with all nutrition interventions, the LSFF of cereal grains is not a stand-alone strategy to improve dietary zinc intake; instead, it should be combined with other interventions to meet the needs of all populations at risk of zinc deficiency, including the most vulnerable. However, investments to strengthen and expand LSFF programmes in which zinc is included as a fortificant hold great potential to enhance dietary zinc intake and improve the population zinc status in countries where zinc deficiency is considered a public health problem.

## Methods

### Estimation of inadequate zinc intake

The analytical methods and model assumptions used to estimate the adequacy of zinc in national food supplies have previously been described extensively^22^. Methodological assumptions from previous models were retained in the analyses reported here to provide consistency of results. In brief, data on the average daily per capita availability of major food commodities (kcal capita^−1^ d^−1^) were obtained from 2018 food balance sheets available from FAO (https://www.fao.org/faostat/en/#data/FBS). Food balance sheets aggregate similar foods into standardized commodities or revert processed foods back to the original commodity, but do not report on the proportion of the average daily per capita availability of the standardized commodities contributed by individual foods or the extent of processing applied to the primary commodity. Thus, all analyses were conducted on the basis of the daily per capita calorific availability of each food commodity (kcal capita^−1^ d^−1^) rather than on a mass basis (g capita^−1^ d^−1^) to estimate the quantity available for consumption more accurately. The total zinc and phytate contents of the daily food supply (mg d^−1^), accounting for food processing methods (for example, extraction rates of milled grains, soaking and fermentation)^22^, were calculated as the sum of the zinc and phytate contents of each food commodity based on a previously published composite nutrient composition database^22^. The following scenarios were modelled (Table 1):

1. Baseline: this model did not account for LSFF programmes as currently implemented and was used to identify only countries at high-risk of inadequate zinc intake.
2. Current programme: this model accounted for LSFF programmes (that is, current country-specific food fortification standards and industry compliance) and the additional extrinsic zinc content contributed by LSFF to the total zinc content of the daily food supply. The GFDx^12^ was used to obtain information on the presence or absence of a mandatory or voluntary food fortification programme in each country and which staple foods were fortified under the aforementioned programme (that is, wheat flour, maize flour and/or rice; GFDx Indicators 1 and 9), whether zinc was included as a fortificant in the programme (standard in mg kg^−1^; GFDx Indicator 6), the percentage of each staple food that was industrially processed (GFDx Indicator 12) and the current percentage compliance with fortification standards (GFDx Indicator 15; Table 2 and Supplementary Table 2). All data were accessed on 22 August 2022. Zinc fortification standards, expressed as milligram Zn per kilogram cereal grain, were converted to milligram Zn per kilocalorie cereal grain to match the established dataset. When data on compliance by product volumes were unavailable, compliance was estimated by one of four proxy indicators (compliance by market share, quality, estimated quality and facilities/samples monitored). In cases where compliance data for mandatory fortification standards were completely unavailable for a specific country, estimated compliance with fortification was calculated as the median compliance with fortification among all other countries on the same continent with mandatory fortification standards for each food commodity. If compliance data for voluntary fortification standards were unavailable for a specific country, compliance was assumed to be zero.

The additional extrinsic zinc content of the daily food supply (mg capita^−1^ d^−1^) contributed by LSFF was calculated as the average daily per capita availability of each food commodity with food fortification standards (kcal capita^−1^ d^−1^) × Zn standard (mg kcal^−1^) × percentage of the food commodity industrially processed × percentage compliance with the fortification standard.

In both model scenarios, the estimated absorbable zinc content of the daily food supply was estimated using the Miller equation, which is a saturation response model of zinc absorption as a function of dietary zinc and phytate^36^. The theoretical mean daily per capita physiological requirement for zinc was calculated using the estimated physiological zinc requirements for absorbed zinc, as reviewed by IZiNCG (ref. ^1^) and based on the age and sex distribution of the population. Population estimates were obtained from the United Nations World Population Prospects 2018 (https://population.un.org/wpp/). The percentage of the mean physiological requirement for zinc available in the food supply was calculated by dividing the estimated absorbable zinc content of the food supply by the calculated theoretical mean daily per capita physiological requirement. The estimated prevalence of inadequate zinc intake was calculated using a method akin to the Institute of Medicine Estimated Average Requirement cut-point method and assuming a 25% inter-individual coefficient of variation. This method of estimating the availability of absorbable zinc in the food supply provides a proxy for dietary zinc intake.

Zinc deficiency was considered a public health problem in LMICs where (1) the national prevalence of inadequate zinc intake (‘baseline’ scenario) was >25% and the prevalence of stunting among children less than 5 years of age was >20% or (2) where the percentage of pre-school children or women of reproductive age with low PZC was ≥20% according to available national surveys^2,14^ (Table 2 and Supplementary Table 1). The data on the prevalence of stunting and low PZCs were obtained from the most recent nationally representative surveys^5,37^.

### Estimation of inadequate zinc intake with modelled scenarios

The total zinc and phytate contents of the daily food supply (mg d^−1^) were recalculated using the following hypothetical scenarios (Table 1):

1. ‘Full compliance’: in countries with mandatory fortification standards, this scenario reflects retaining the current standards while compliance with the fortification standard is increased to 100% of industrially processed food commodities. In countries with no mandatory fortification standards, or mandatory fortification standards that do not include zinc, this model is the same as the ‘current programme’ scenario.
2. ‘Aligned standards’: in countries with mandatory fortification standards, this scenario reflects adding zinc to the mandatory standard (if it is not already included) or aligning the standard to reflect current international zinc fortification guidelines (Extended Data Table 1). The standard applied reflects the estimated per capita availability (g capita^−1^ d^−1^) of each staple food and the estimated extraction rate for wheat and maize flour^22^. In countries with no mandatory fortification standards, this model is the same as the ‘current programme’ scenario.
3. ‘New/aligned standards with full compliance’: in all countries, this scenario reflects a mandatory fortification standard that includes zinc at levels reflecting current zinc fortification guidelines as well as 100% compliance of industrially processed food commodities with the fortification standard. Wheat flour, maize flour and rice were modelled separately and combined. In addition, we ran a sensitivity analysis of the combined programme, retaining the aforementioned assumptions but reducing compliance to 85% (if current compliance with existing programmes was already >85%, current compliance was retained).

The estimated absorbable zinc content of the daily food supply, the percentage of the mean physiological requirement for zinc available in the food supply and the estimated prevalence of inadequate zinc intake were then recalculated for each scenario using the methods detailed above.

Although not a primary objective of this analysis, estimates from each of the hypothetical scenarios detailed above were also generated for all countries with available data to provide information to relevant stakeholders (Supplementary Table 3).

### Statistical analyses

Regional classifications were based on the reporting regions of the Global Burden of Diseases, Injuries and Risk Factors 2010 Study^38^. The regional and global data are for 174 countries with available data (that is, data on national food supply, LSFF programmes and population) and are weighted by national population size. For regional and global estimates, the ‘full compliance’, ‘aligned standards’ and ‘new/aligned standards with full compliance’ scenarios were applied only to countries where zinc deficiency was considered a public health problem; the estimates for all other countries were obtained according to the ‘current programme’ scenario. All statistical analyses were completed using the SAS System for Windows (release 9.4, SAS Institute). The data are presented as the mean ± s.d. or median (first and third quartiles), unless otherwise noted.

### Reporting summary

Further information on research design is available in the Nature Portfolio Reporting Summary linked to this article.

### Supplementary information

Supplementary Information Supplementary Tables 1–3.

Reporting Summary

### Source data

Source Data Fig. 1 Statistical source data.

## Figures and Tables

**Extended Data Table 1:**
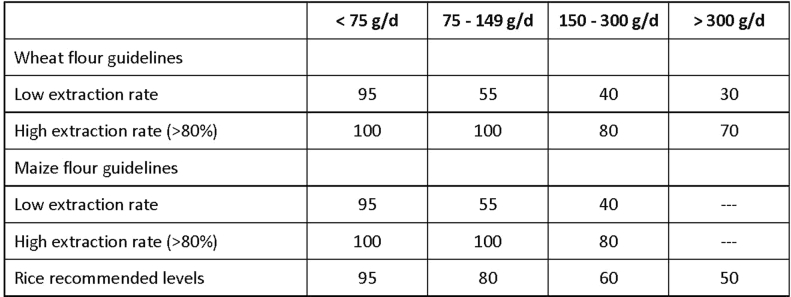
Level of zinc to consider for adding to fortified wheat flour, fortified maize flour and corn meal, or rice when each is the only micronutrient intervention, based on extraction rate and estimated per capita consumption (mg/kg)

Levels as recommended by WHO guidelines for the fortification of wheat flour^17^ and maize flour^18^, and recommendations for the fortification of rice^19^. These estimated target levels consider only one staple as the main fortification vehicle in a public health programme. If other LSFF programmes are implemented effectively, these suggested fortification levels may need to be adjusted downwards as needed. For estimated per capita consumption of maize flour > 300 g/d, wheat flour guidelines were used in analyses.

## Data Availability

Data on the average daily per capita availability of major food commodities (kcal capita^−1^ d^−1^) were obtained from food balance sheets (2018) available from the FAO (https://www.fao.org/faostat/en/#data/FBS)^39^. Information on fortification programmes and standards, percentage of cereal grains industrially processed and reported compliance were obtained from the GFDx (https://fortificationdata.org/full-gfdx-datasets/)^12^. Population estimates were obtained from the United Nations World Population Prospects 2018 (https://population.un.org/wpp/). The nutrient composition data and extraction and processing estimates have been published previously and are available online (10.1371/journal.pone.0050565)^22^. Compiled datasets used in the analytic code for these analyses are available on the Open Science Framework (https://osf.io/58wst/)^40^. Source data are provided with this paper.

## Code availability

The analyses were performed using SAS System for Windows (release 9.4). The analytic code for these analyses is available on the Open Science Framework (https://osf.io/58wst/)^40^.
