# Supplementary material for: Mandatory large-scale food fortification programmes can reduce the estimated prevalence of inadequate zinc intake by up to 50% globally
Source: Nat Food. 2024 Jun 19;5(7):625–37. doi: 10.1038/s43016-024-00997-w (PMC11272573; doi:10.1038/s43016-024-00997-w)
Supplement: Supplementary file 1 — Supplementary Tables 1–3. [file 43016_2024_997_MOESM1_ESM.pdf]

nature food

Supplementary information https://doi.org/10.1038/s43016-024-00997-w

Mandatory large-scale food fortification programmes can reduce the estimated prevalence of inadequate zinc intake by up to 50% globally

In the format provided by the authors and unedited

Supplementary Table 1. Population-level risk of zinc deficiency, based on proxy indicators (estimated prevalence of inadequate zinc intake and prevalence of stunting among children under 5) and low plasma zinc concentrations among children < 5 years of age and women of reproductive age

Country	Zinc deficiency as a public health problem[1]	Estimated prevalence of inadequate zinc intake ("*baseline*"), %[2]	Stunting, %	Stunting data, year	% low PZC in children	% low PZC in women	PZC data, year
**South Asia**							
Afghanistan	yes	20.9	38.2	2018	15.1	23.4	2013
Bangladesh	yes	24.8	28	2019	44.6	57.3	2012
India	yes	29.2	34.7	2017	18.9	28.4	2018
Nepal	yes	20.2	31.5	2019	20.7	24.3	2016
Pakistan	yes	19.7	37.6	2018	18.6	22.1	2018
**Sub-Saharan Africa**							
Angola	no	18.3	37.6	2015			
Benin	no	16.0	32.2	2018			
Botswana	yes	27.4	28.9	2007			
Burkina Faso	yes	35.2	24.9	2020			
Burundi	yes	38.3	50.9	2020			
Cabo Verde	no	20.6	21.4	1994			
Cameroon	yes	26.0	28.9	2018	82.6	81.6	2009
Central African Republic	no	10.8	40.2	2019			
Chad	yes	33.1	31.1	2021			
Comoros	yes	28.2	31.1	2012			
Congo	no	14.3	21.2	2014			
Côte d'Ivoire	yes	28.3	21.6	2016			
Democratic Republic of the Congo	yes	38.8	41.8	2017			
Djibouti	no	21.7	20.9	2019			
Eswatini	yes	25.2	25.5	2014			
Ethiopia	yes	10.2	36.8	2019	35	33.8	2015
Gabon	no	13.0	17	2012			
Gambia	no	30.6	17.5	2020			
Ghana	no	19.1	17.5	2017			
Guinea	no	15.0	30.3	2018			
Guinea-Bissau	no	22.4	28.1	2019			
Kenya	yes	25.0	26.2	2014	81.6	79.9	2011
Lesotho	yes	34.2	34.6	2018			
Liberia	no	22.5	29.8	2019			
Madagascar	no	20.7	39.8	2021			
Malawi	yes	26.9	34.9	2020	60.4	62.5	2016
Mali	no	18.5	22.1	2021			
Mauritania	no	11.0	17.4	2021			
Mauritius	no	16.3	13.6	1995			
Mozambique	yes	28.5	37.5	2020			
Namibia	no	21.4	22.7	2013			
Niger	no	21.7	44.4	2021			
Nigeria	yes	17.7	31.5	2020	20	43.8	2001
Rwanda	yes	28.9	33.1	2020			
Sao Tome and Principe	no	30.1	11.7	2019			
Senegal	yes	24.4	17.9	2019	50	59	2010
Seychelles	no	13.1	7.9	2012			
Sierra Leone	no	22.6	26.3	2021			

Country	Zinc deficiency as a public health problem[1]	Estimated prevalence of inadequate zinc intake (“*baseline*”), %[2]	Stunting, %	Stunting data, year	% low PZC in children	% low PZC in women	PZC data, year
South Africa	yes	15.8	21.4	2017	51		2005
Sudan	no	10.7	38.2	2014			
Togo	no	20.3	23.8	2017			
Uganda	no	24.4	25.4	2020			
United Republic of Tanzania	yes	26.3	31.8	2018			
Zambia	yes	36.9	34.6	2018			
Zimbabwe	yes	45.4	23.5	2019			
**Central Asia, North Africa, and Middle East**							
Algeria	no	12.3	9.8	2019			
Armenia	no	12.1	9.4	2016			
Azerbaijan	no	17.6	17.8	2013	10.7		2013
Egypt	no	9.5	22.3	2014			
Georgia	no	24.3	5.8	2018			
Iran (Islamic Republic of)	yes	23.3	4.8	2017	19.1	28	2015
Iraq	no	20.2	12.6	2018			
Jordan	no	18.9	7.4	2019			
Kazakhstan	no	3.5	8	2015			
Kuwait	no	15.1	6.4	2017			
Kyrgyzstan	no	6.4	11.8	2018			
Lebanon	no	21.8	16.5	2004			
Libyan Arab Jamahiriya	no	19.2	38.1	2014			
Mongolia	no	2.6	9.4	2018			
Morocco	no	16.5	15.1	2017			
Oman	no	17.6	11.4	2017			
Saudi Arabia	no	25.7	9.3	2004			
Syrian Arab Republic	no	22.8	27.9	2010			
Tajikistan	no	14.9	17.5	2017			
Tunisia	no	14.7	8.4	2018			
Turkey	no	17.7	6	2018			
Turkmenistan	no	3.6	7.2	2019			
United Arab Emirates	no	22.4					
Uzbekistan	no	4.4	10.8	2017			
Yemen	yes	25.9	46.4	2013			
**Central and Andean Latin America and Caribbean**							
Antigua and Barbuda	no	8.1					
Bahamas	no	6.5					
Barbados	no	10.2	7.7	2012			
Belize	no	16.2	15	2015			
Bolivia (Plurinational State of)	no	7.9	16.1	2016			
Colombia	yes	9.3	12.7	2016	43		2010
Costa Rica	yes	14.0	9	2018	23.9		2009
Cuba	no	12.9	7.1	2019			
Dominican Republic	no	12.0	6.7	2019			
Ecuador	yes	11.5	23	2019	28	56	2012
El Salvador	no	29.7	13.6	2014			
Grenada	no	9.3					
Guatemala	yes	28.5	46.7	2015	13.3	18.3	2016
Guyana	no	9.5	9.1	2019			

Country	Zinc deficiency as a public health problem[1]	Estimated prevalence of inadequate zinc intake ("*baseline*"), %[2]	Stunting, %	Stunting data, year	% low PZC in children	% low PZC in women	PZC data, year
Haiti	yes	29.1	21.9	2017			
Honduras	no	31.6	18.7	2019			
Jamaica	no	14.2	4.6	2018			
Mexico	yes	14.9	13.9	2020	26.6	33.8	2006
Nicaragua	no	33.3	17.3	2012			
Panama	no	14.4	15.9	2019			
Peru	no	16.9	11.4	2020			
Saint Lucia	no	7.1	2.5	2012			
Saint Vincent and the Grenadines	no	8.4					
Suriname	no	16.8	8.3	2018			
Trinidad and Tobago	no	13.2	9.2	2011			
Venezuela (Bolivarian Republic of)	no	23.4	13.4	2009			
**East and Southeast Asia and Pacific**							
Cambodia	yes	19.2	32.4	2014	67.5	62.8	2014
Democratic People's Republic of Korea	no	41.8	19.1	2017			
Fiji	no	13.8	7.5	2004		0	2010
French Polynesia	no	7.1					
Indonesia	yes	26.4	30.8	2018			
Kiribati	no	25.3	15.2	2018			
Lao People's Democratic Republic	no	11.1	33.1	2017			
Malaysia	no	12.7	21.8	2019			
Maldives	yes	25.9	15.3	2017	16	27	2007
Myanmar	no	10.4	28.7	2018			
New Caledonia	no	7.1					
Papua New Guinea	no	13.4	49.5	2010			
Philippines	yes	11.2	28.8	2019	17.9	28.4	2013
Samoa	no	7.6					
Solomon Islands	no	23.9	31.7	2015			
Sri Lanka	no	35.2	17.3	2016	5.1		2012
Thailand	no	17.7	13.4	2019			
Timor-Leste	yes	21.5	46.7	2020	60.3		2013
Vanuatu	no	14.1	28.9	2013			
Viet Nam	yes	8.0	19.6	2020	51.9	67.2	2010
**Central and Eastern Europe**							
Albania	no	5.7	11.3	2017			
Belarus	no	7.3					
Bosnia and Herzegovina	no	11.8	8.9	2012			
Bulgaria	no	13.0	7	2014			
Croatia	no	7.3					
Czechia	no	8.8	2.7	2001			
Estonia	no	8.0	1.2	2014			
Hungary	no	6.9					
Latvia	no	9.7					
Lithuania	no	6.7					
Montenegro	no	4.9	7.2	2018			
North Macedonia	no	13.5	4.3	2019			

Country	Zinc deficiency as a public health problem[1]	Estimated prevalence of inadequate zinc intake (“*baseline*”), %[2]	Stunting, %	Stunting data, year	% low PZC in children	% low PZC in women	PZC data, year
Poland	no	8.5	2.6	2014			
Republic of Moldova	no	17.7	6.4	2012			
Romania	no	7.8	12.8	2002			
Russian Federation	no	6.1					
Serbia	no	8.5	5.4	2019			
Slovakia	no	14.0					
Slovenia	no	13.5					
Ukraine	no	11.1	22.9	2000			
**China**							
China	no	6.6	4.8	2017			
**Southern and Tropical Latin America**							
Argentina	no	2.0	8.7	2018			
Brazil	no	6.2	7	2007			
Chile	no	5.5	1.8	2014			
Paraguay	no	15.5	5.6	2016			
Uruguay	no	5.7	6.9	2018			
**High-income**							
Australia	no	4.6	2	2007			
Austria	no	8.2				18.1	2012
Belgium	no	7.4	1.6	2014			
Canada	no	9.3					
Cyprus	no	10.1					
Denmark	no	6.4					
Finland	no	5.1					
France	no	5.1					
Germany	no	9.3	1.7	2016			
Greece	no	8.6	1.5	2003			
Iceland	no	2.8					
Ireland	no	4.6					
Israel	no	4.9					
Italy	no	7.3					
Japan	no	17.6	7.1	2010			
Luxembourg	no	5.6					
Malta	no	8.0					
Netherlands	no	6.2	1.5	2009			
New Zealand	no	7.1					
Norway	no	6.5					
Portugal	no	6.6	3.2	2016			
Republic of Korea	no	10.0	2.5	2009			
Spain	no	6.7					
Sweden	no	6.3					
Switzerland	no	5.4					
United Kingdom of Great Britain and Northern Ireland	no	5.9					
United States of America	no	5.8	3.4	2018			

[1]Zinc deficiency was considered to be a public health problem in low- and middle-income countries where the percentage of the population at risk of inadequate zinc intake due to inadequate zinc in the national food supply

("*baseline*" scenario) was >25% and the prevalence of stunting among children less than 5 years of age was >20%[1], or where the percentage of pre-school children or women of reproductive age with low PZC was ≥20% according to the most recently available national survey.

[2]Estimated country-specific prevalence of inadequate zinc intake, not accounting for current LSFF programs (*"baseline"* scenario). Data are based on the 2018 FAO food balance sheet data, a composite nutrient composition database, IZiNCG physiological requirements, the Miller Equation to estimate zinc absorption[38] and an assumed 25% inter-individual variation in zinc intake.

Supplementary Table 2. Current fortification standards in all countries with available data[1]

Country	Wheat flour fortification legislation	Wheat flour, zinc standard (mg/kg)	Wheat flour, % industrially processed	Wheat flour, % compliance with fortification	Maize flour fortification legislation	Maize flour, zinc standard (mg/kg)	Maize flour, % industrially processed	Maize flour, % compliance with fortification	Rice fortification legislation	Rice, zinc standard (mg/kg)	Rice, % industrially processed	Rice, % compliance with fortification
**South Asia**												
Afghanistan	mandatory	50	63	71.2	no or unk	NA	0	NA	no or unk	NA	55	NA
Bangladesh	voluntary	27	78	-	no or unk	NA	0	NA	voluntary	40	28	1
India	voluntary	12.5	32	-	no or unk	NA	0	NA	voluntary	12.5	50	-
Nepal	mandatory	0	25	36.8	no or unk	NA	99	NA	no or unk	NA	27	NA
Pakistan	no or unk	NA	32	NA	no or unk	NA	0	NA	no or unk	NA	90	NA
**Sub-Saharan Africa**												
Angola	no or unk	NA	100	NA	no or unk	NA	0	NA	no or unk	NA	100	NA
Benin	mandatory	0	100	-	no or unk	NA	0	NA	no or unk	NA	84	NA
Botswana	no or unk	NA	100	NA	no or unk	NA	98	NA	no or unk	NA	100	NA
Burkina Faso	mandatory	0	100	61.5	no or unk	NA	0	NA	no or unk	NA	72	NA
Burundi	mandatory	88	40	-	mandatory	49	0	-	no or unk	NA	30	NA
Cabo Verde	mandatory	0	100	98	no or unk	NA	0	NA	no or unk	NA	100	NA
Cameroon	mandatory	95	100	100	no or unk	NA	100	NA	no or unk	NA	78	NA
Central African Republic	no or unk	NA	100	NA	no or unk	NA	0	NA	no or unk	NA	0	NA
Chad	mandatory	0	38	0	mandatory	0	0	0	no or unk	NA	7	NA
Comoros	no or unk	NA	100	NA	no or unk	NA	0	NA	no or unk	NA	100	NA
Congo	mandatory	0	100	-	no or unk	NA	0	NA	no or unk	NA	100	NA
Côte d'Ivoire	mandatory	0	100	99	no or unk	NA	100	NA	no or unk	NA	51.12	NA
Democratic Republic of the Congo	no or unk	NA	100	NA	no or unk	NA	5	NA	no or unk	NA	5	NA
Djibouti	mandatory	40	100	-	no or unk	NA	0	NA	no or unk	NA	100	NA
Eswatini	voluntary	20	100	-	no or unk	NA	0	NA	no or unk	NA	100	NA
Ethiopia	mandatory	80	38.85	0	no or unk	NA	30	NA	no or unk	NA	0	NA
Gabon	no or unk	NA	100	NA	no or unk	NA	100	NA	no or unk	NA	79	NA
Gambia	mandatory	0	100	50	no or unk	NA	0	NA	no or unk	NA	100	NA
Ghana	mandatory	28.3	100	100	no or unk	NA	0	NA	no or unk	NA	92	NA
Guinea	mandatory	0	100	-	no or unk	NA	0	NA	no or unk	NA	67	NA
Guinea-Bissau	no or unk	NA	100	NA	no or unk	NA	0	NA	no or unk	NA	56	NA
Kenya	mandatory	40	100	100	mandatory	30	51	0	no or unk	NA	83	NA
Lesotho	mandatory	0	100	66	mandatory	0	71	0	no or unk	NA	100	NA
Liberia	mandatory	95	100	100	no or unk	NA	0	NA	no or unk	NA	64	NA
Madagascar	no or unk	NA	100	NA	no or unk	NA	0	NA	no or unk	NA	21	NA
Malawi	mandatory	80	100	20	mandatory	40	15	40	no or unk	NA	6	NA
Mali	mandatory	0	100	-	no or unk	NA	0	NA	no or unk	NA	52	NA

Country	Wheat flour fortification legislation	Wheat flour, zinc standard (mg/kg)	Wheat flour, % industrially processed	Wheat flour, % compliance with fortification	Maize flour fortification legislation	Maize flour, zinc standard (mg/kg)	Maize flour, % industrially processed	Maize flour, % compliance with fortification	Rice fortification legislation	Rice, zinc standard (mg/kg)	Rice, % industrially processed	Rice, % compliance with fortification
Mauritania	mandatory	0	100	55	no or unk	NA	0	NA	no or unk	NA	30	NA
Mauritius	no or unk	NA	95	NA	no or unk	NA	0	NA	no or unk	NA	100	NA
Mozambique	mandatory	30	100	60	mandatory	20	30	70	no or unk	NA	68	NA
Namibia	no or unk	NA	98	NA	no or unk	NA	0	NA	no or unk	NA	91	NA
Niger	mandatory	0	100	80	no or unk	NA	0	NA	no or unk	NA	100	NA
Nigeria	mandatory	50	96	74	mandatory	50	0	80	no or unk	NA	80	NA
Rwanda	mandatory	60	60	10	mandatory	49	35	40	no or unk	NA	40	NA
Sao Tome and Principe	no or unk	NA	100	NA	no or unk	NA	0	NA	no or unk	NA	100	NA
Senegal	mandatory	0	72	96	no or unk	NA	0	NA	no or unk	NA	100	NA
Seychelles	no or unk	NA	100	NA	no or unk	NA	0	NA	no or unk	NA	0	NA
Sierra Leone	voluntary	28.3	100	-	no or unk	NA	13	NA	no or unk	NA	38	NA
South Africa	mandatory	15	100	80	mandatory	15	75	83.4	no or unk	NA	100	NA
Sudan	voluntary	40	80	40	no or unk	NA	75	NA	no or unk	NA	0	NA
Togo	mandatory	55	70	100	no or unk	NA	10	NA	no or unk	NA	100	NA
Uganda	mandatory	60	100	100	mandatory	30	42	6	no or unk	NA	36	NA
United Republic of Tanzania	mandatory	40	99.5	86	mandatory	22.5	45	7	no or unk	NA	0	NA
Zambia	voluntary	0	100	-	no or unk	NA	35	NA	no or unk	NA	37	NA
Zimbabwe	mandatory	40	80	40	mandatory	40	38	5	no or unk	NA	100	NA
**Central Asia, North Africa and Middle East**												
Algeria	no or unk	NA	100	NA	no or unk	NA	0	NA	no or unk	NA	100	NA
Armenia	no or unk	NA	80	NA	no or unk	NA	100	NA	no or unk	NA	100	NA
Azerbaijan	no or unk	NA	70	NA	no or unk	NA	100	NA	no or unk	NA	100	NA
Egypt	no or unk	NA	92	NA	no or unk	NA	0	NA	no or unk	NA	100	NA
Georgia	no or unk	NA	100	NA	no or unk	NA	100	NA	no or unk	NA	100	NA
Iran (Islamic Republic of)	mandatory	0	100	-	no or unk	NA	0	NA	no or unk	NA	60	NA
Iraq	voluntary	0	100	-	no or unk	NA	0	NA	no or unk	NA	84	NA
Jordan	mandatory	20.08	100	90	no or unk	NA	100	NA	no or unk	NA	99	NA
Kazakhstan	mandatory	25	100	41	no or unk	NA	100	NA	no or unk	NA	100	NA
Kuwait	voluntary	0	100	-	no or unk	NA	0	NA	no or unk	NA	100	NA
Kyrgyzstan	mandatory	0	85	-	no or unk	NA	100	NA	no or unk	NA	100	NA
Lebanon	no or unk	NA	100	NA	no or unk	NA	0	NA	no or unk	NA	100	NA
Libyan Arab Jamahiriya	no or unk	NA	100	NA	no or unk	NA	0	NA	no or unk	NA	0	NA
Mongolia	mandatory	18.7	90	-	no or unk	NA	0	NA	no or unk	NA	0	NA
Morocco	mandatory	0	100	70	no or unk	NA	0	NA	no or unk	NA	76	NA

Country	Wheat flour fortification legislation	Wheat flour, zinc standard (mg/kg)	Wheat flour, % industrially processed	Wheat flour, % compliance with fortification	Maize flour fortification legislation	Maize flour, zinc standard (mg/kg)	Maize flour, % industrially processed	Maize flour, % compliance with fortification	Rice fortification legislation	Rice, zinc standard (mg/kg)	Rice, % industrially processed	Rice, % compliance with fortification
Oman	mandatory	0	100	100	no or unk	NA	0	NA	no or unk	NA	100	NA
Saudi Arabia	voluntary	0	100	-	no or unk	NA	100	NA	no or unk	NA	100	NA
Syrian Arab Republic	no or unk	NA	80	NA	no or unk	NA	0	NA	no or unk	NA	0	NA
Tajikistan	mandatory	0	95	0	no or unk	NA	100	NA	no or unk	NA	100	NA
Tunisia	no or unk	NA	100	NA	no or unk	NA	90	NA	no or unk	NA	100	NA
Turkey	no or unk	NA	100	NA	no or unk	NA	0	NA	no or unk	NA	100	NA
Turkmenistan	mandatory	0	100	95	no or unk	NA	100	NA	no or unk	NA	100	NA
United Arab Emirates	voluntary	0	100	-	no or unk	NA	90	NA	no or unk	NA	100	NA
Uzbekistan	mandatory	22.5	80	1	no or unk	NA	0	NA	no or unk	NA	100	NA
Yemen	mandatory	0	100	-	no or unk	NA	0	NA	no or unk	NA	100	NA
**Central and Andean Latin America and Caribbean**												
Antigua and Barbuda	mandatory	0	100	-	no or unk	NA	0	NA	no or unk	NA	100	NA
Bahamas	mandatory	0	100	-	no or unk	NA	0	NA	no or unk	NA	100	NA
Barbados	mandatory	0	100	-	no or unk	NA	0	NA	no or unk	NA	100	NA
Belize	mandatory	0	100	100	no or unk	NA	0	NA	voluntary	0	100	0
Bolivia (Plurinational State of)	mandatory	0	80	-	no or unk	NA	0	NA	no or unk	NA	8	NA
Colombia	mandatory	0	100	81.3	no or unk	NA	0	NA	no or unk	NA	80	NA
Costa Rica	mandatory	0	100	100	mandatory	0	100	100	mandatory	7.5	100	100
Cuba	mandatory	0	100	-	no or unk	NA	0	NA	no or unk	NA	70	NA
Dominican Republic	mandatory	0	100	100	voluntary	0	0	0	no or unk	NA	0	NA
Ecuador	mandatory	0	100	-	no or unk	NA	0	NA	no or unk	NA	0	NA
El Salvador	mandatory	0	100	-	mandatory	0	0	-	no or unk	NA	100	NA
Grenada	mandatory	0	100	-	no or unk	NA	0	NA	no or unk	NA	100	NA
Guatemala	mandatory	0	100	100	mandatory	15	0	100	no or unk	NA	94	NA
Guyana	mandatory	0	100	100	no or unk	NA	0	NA	no or unk	NA	0	NA
Haiti	mandatory	0	100	75	no or unk	NA	10	NA	no or unk	NA	90	NA
Honduras	mandatory	0	100	100	no or unk	NA	0	NA	no or unk	NA	98	NA
Jamaica	mandatory	0	100	-	no or unk	NA	0	NA	no or unk	NA	100	NA
Mexico	mandatory	40	100	0	mandatory	40	40	3.1	no or unk	NA	100	NA
Nicaragua	mandatory	0	100	-	no or unk	NA	0	NA	mandatory	25	31	-
Panama	mandatory	0	100	100	no or unk	NA	0	NA	mandatory	25	100	100
Peru	mandatory	0	90	93.2	no or unk	NA	0	NA	mandatory	32	26	0
Saint Lucia	mandatory	0	100	-	no or unk	NA	0	NA	no or unk	NA	100	NA
Saint Vincent and the Grenadines	mandatory	0	100	-	no or unk	NA	0	NA	no or unk	NA	0	NA
Suriname	mandatory	0	100	-	no or unk	NA	0	NA	no or unk	NA	0	NA

Country	Wheat flour fortification legislation	Wheat flour, zinc standard (mg/kg)	Wheat flour, % industrially processed	Wheat flour, % compliance with fortification	Maize flour fortification legislation	Maize flour, zinc standard (mg/kg)	Maize flour, % industrially processed	Maize flour, % compliance with fortification	Rice fortification legislation	Rice, zinc standard (mg/kg)	Rice, % industrially processed	Rice, % compliance with fortification
Trinidad and Tobago	mandatory	0	100	-	no or unk	NA	0	NA	no or unk	NA	100	NA
Venezuela (Bolivarian Republic of)	mandatory	0	100	-	mandatory	0	0	-	voluntary	0	73	-
**East and Southeast Asia and Pacific**												
Cambodia	no or unk	NA	100	NA	no or unk	NA	3	NA	no or unk	NA	0	NA
Democratic People's Republic of Korea	no or unk	NA	100	NA	no or unk	NA	0	NA	no or unk	NA	100	NA
Fiji	mandatory	30	100	100	no or unk	NA	0	NA	no or unk	NA	100	NA
French Polynesia	no or unk	NA	100	NA	no or unk	NA	0	NA	no or unk	NA	100	NA
Indonesia	mandatory	30	100	87	no or unk	NA	0	NA	no or unk	NA	7	NA
Kiribati	mandatory	30	100	-	no or unk	NA	0	NA	no or unk	NA	100	NA
Lao People's Democratic Republic	no or unk	NA	100	NA	no or unk	NA	0	NA	no or unk	NA	15	NA
Malaysia	voluntary	0	100	-	no or unk	NA	0	NA	no or unk	NA	37	NA
Maldives	no or unk	NA	100	NA	no or unk	NA	0	NA	no or unk	NA	100	NA
Myanmar	no or unk	NA	100	NA	no or unk	NA	0	NA	voluntary	50	1.6	1
New Caledonia	no or unk	NA	100	NA	no or unk	NA	0	NA	no or unk	NA	100	NA
Papua New Guinea	no or unk	NA	95	NA	no or unk	NA	0	NA	mandatory	0	90	76
Philippines	mandatory	0	100	-	no or unk	NA	0	NA	mandatory	0	29	-
Samoa	mandatory	30	100	-	no or unk	NA	0	NA	voluntary	0	100	-
Solomon Islands	mandatory	30	100	-	no or unk	NA	100	NA	mandatory	45	95	100
Sri Lanka	no or unk	NA	100	NA	no or unk	NA	0	NA	no or unk	NA	34	NA
Thailand	no or unk	NA	100	NA	no or unk	NA	0	NA	no or unk	NA	70	NA
Timor-Leste	no or unk	NA	100	NA	no or unk	NA	0	NA	no or unk	NA	100	NA
Vanuatu	no or unk	NA	100	NA	no or unk	NA	0	NA	no or unk	NA	10	NA
Viet Nam	mandatory	101.3	100	-	no or unk	NA	0	NA	no or unk	NA	20	NA
**Central and Eastern Europe**												
Albania	no or unk	NA	100	NA	no or unk	NA	100	NA	no or unk	NA	100	NA
Belarus	no or unk	NA	62	NA	no or unk	NA	100	NA	no or unk	NA	100	NA
Bosnia and Herzegovina	no or unk	NA	100	NA	no or unk	NA	100	NA	no or unk	NA	100	NA
Bulgaria	no or unk	NA	100	NA	no or unk	NA	100	NA	no or unk	NA	100	NA
Croatia	no or unk	NA	100	NA	no or unk	NA	100	NA	no or unk	NA	100	NA
Czechia	no or unk	NA	100	NA	no or unk	NA	100	NA	no or unk	NA	100	NA
Estonia	no or unk	NA	100	NA	no or unk	NA	100	NA	no or unk	NA	100	NA
Hungary	no or unk	NA	100	NA	no or unk	NA	100	NA	no or unk	NA	100	NA
Latvia	no or unk	NA	100	NA	no or unk	NA	100	NA	no or unk	NA	100	NA
Lithuania	no or unk	NA	100	NA	no or unk	NA	100	NA	no or unk	NA	100	NA

Country	Wheat flour fortification legislation	Wheat flour, zinc standard (mg/kg)	Wheat flour, % industrially processed	Wheat flour, % compliance with fortification	Maize flour fortification legislation	Maize flour, zinc standard (mg/kg)	Maize flour, % industrially processed	Maize flour, % compliance with fortification	Rice fortification legislation	Rice, zinc standard (mg/kg)	Rice, % industrially processed	Rice, % compliance with fortification
Montenegro	no or unk	NA	100	NA	no or unk	NA	100	NA	no or unk	NA	100	NA
North Macedonia	no or unk	NA	100	NA	no or unk	NA	100	NA	no or unk	NA	100	NA
Poland	no or unk	NA	100	NA	no or unk	NA	100	NA	no or unk	NA	100	NA
Republic of Moldova	mandatory	0	91.8	0.6	no or unk	NA	100	NA	no or unk	NA	100	NA
Romania	no or unk	NA	75	NA	no or unk	NA	80	NA	no or unk	NA	100	NA
Russian Federation	no or unk	NA	100	NA	no or unk	NA	100	NA	no or unk	NA	100	NA
Serbia	no or unk	NA	65	NA	no or unk	NA	100	NA	no or unk	NA	100	NA
Slovakia	no or unk	NA	100	NA	no or unk	NA	100	NA	no or unk	NA	100	NA
Slovenia	no or unk	NA	100	NA	no or unk	NA	100	NA	no or unk	NA	100	NA
Ukraine	no or unk	NA	90	NA	no or unk	NA	0	NA	no or unk	NA	78	NA
**China**												
China	voluntary	25	100	-	no or unk	NA	0	NA	no or unk	NA	78	NA
**Southern and Tropical Latin America**												
Argentina	mandatory	0	100	100	no or unk	NA	100	NA	no or unk	NA	100	NA
Brazil	mandatory	0	100	81	mandatory	0	100	33	no or unk	NA	100	NA
Chile	mandatory	0	100	100	no or unk	NA	0	NA	no or unk	NA	100	NA
Paraguay	mandatory	0	100	100	no or unk	NA	0	NA	no or unk	NA	100	NA
Uruguay	mandatory	0	100	28	no or unk	NA	0	NA	no or unk	NA	40	NA
**High-income**												
Australia	mandatory	0	100	80	no or unk	NA	100	NA	no or unk	NA	100	NA
Austria	no or unk	NA	100	NA	no or unk	NA	100	NA	no or unk	NA	100	NA
Belgium	no or unk	NA	100	NA	no or unk	NA	100	NA	no or unk	NA	100	NA
Canada	mandatory	0	100	95	no or unk	NA	0	NA	voluntary	0	100	0
Cyprus	no or unk	NA	100	NA	no or unk	NA	100	NA	no or unk	NA	100	NA
Denmark	no or unk	NA	100	NA	no or unk	NA	0	NA	no or unk	NA	100	NA
Finland	no or unk	NA	100	NA	no or unk	NA	100	NA	no or unk	NA	100	NA
France	no or unk	NA	100	NA	no or unk	NA	0	NA	no or unk	NA	100	NA
Germany	no or unk	NA	100	NA	no or unk	NA	0	NA	no or unk	NA	100	NA
Greece	no or unk	NA	100	NA	no or unk	NA	0	NA	no or unk	NA	100	NA
Iceland	no or unk	NA	100	NA	no or unk	NA	100	NA	no or unk	NA	100	NA
Ireland	no or unk	NA	100	NA	no or unk	NA	100	NA	no or unk	NA	100	NA
Israel	no or unk	NA	100	NA	no or unk	NA	0	NA	no or unk	NA	100	NA
Italy	no or unk	NA	100	NA	no or unk	NA	0	NA	no or unk	NA	100	NA
Japan	no or unk	NA	100	NA	no or unk	NA	0	NA	no or unk	NA	100	NA
Luxembourg	no or unk	NA	100	NA	no or unk	NA	100	NA	no or unk	NA	100	NA
Malta	no or unk	NA	100	NA	no or unk	NA	100	NA	no or unk	NA	100	NA
Netherlands	no or unk	NA	100	NA	no or unk	NA	0	NA	no or unk	NA	100	NA
New Zealand	mandatory	0	100	38	no or unk	NA	100	NA	no or unk	NA	100	NA

Country	Wheat flour fortification legislation	Wheat flour, zinc standard (mg/kg)	Wheat flour, % industrially processed	Wheat flour, % compliance with fortification	Maize flour fortification legislation	Maize flour, zinc standard (mg/kg)	Maize flour, % industrially processed	Maize flour, % compliance with fortification	Rice fortification legislation	Rice, zinc standard (mg/kg)	Rice, % industrially processed	Rice, % compliance with fortification
Norway	no or unk	NA	100	NA	no or unk	NA	100	NA	no or unk	NA	100	NA
Portugal	no or unk	NA	100	NA	no or unk	NA	0	NA	no or unk	NA	100	NA
Republic of Korea	no or unk	NA	100	NA	no or unk	NA	0	NA	no or unk	NA	0	NA
Spain	no or unk	NA	100	NA	no or unk	NA	0	NA	no or unk	NA	100	NA
Sweden	no or unk	NA	100	NA	no or unk	NA	0	NA	no or unk	NA	100	NA
Switzerland	no or unk	NA	100	NA	no or unk	NA	0	NA	no or unk	NA	100	NA
United Kingdom of Great Britain and Northern Ireland	mandatory	0	99.9	90	no or unk	NA	100	NA	no or unk	NA	100	NA
United States of America	mandatory	0	100	-	mandatory	0	100	-	mandatory	0	100	70

NA, not applicable; unk, unknown

[1]Global Fortification Data Exchange (GFDx)[13] was used to obtain information on the presence or absence of a mandatory or voluntary food fortification program in each country and which staple foods were fortified under the aforementioned program (i.e., wheat flour, maize flour, and/or rice) (Indicators 1 and 9), whether zinc was included as a fortificant in the program (standard mg/kg) (Indicator 6), the percent of each staple food that was industrially processed (Indicator 12), and the current percent compliance with fortification standards (Indicator 15). Data are as reported in the GFDx and are the latest available data (regardless of year). Data on compliance with fortification are not available for all countries. If data were unavailable (denoted in the table as "-"), estimated compliance with mandatory fortification for all modeled scenarios was calculated as the median compliance with fortification among all other countries on the same continent with mandatory fortification standards for each food commodity; compliance with voluntary fortification was assumed to be zero.

Supplementary Table 3. Country-specific estimated prevalence of inadequate zinc intake in the population among all countries with available data[1]

Country	Zinc deficiency as a public health problem	Current program[2], %	Full compliance[3], %	Aligned standards [4], %	New/ aligned standards with full compliance Wheat Flour only [5], %	New/ aligned standards with full compliance, Maize Flour only[5], %	New/ aligned standards with full compliance, Rice only[5], %	New/ aligned standards with full compliance, Combined[5], %
**South Asia**								
Afghanistan	yes	4.9	3.3	3.4	2.4	4.8	3.9	2.1
Bangladesh	yes	24.4	24.4	24.4	9.3	24.4	5.5	3.4
India	yes	29.2	29.2	29.2	12.7	29.2	9.6	5.7
Nepal	yes	20.2	20.2	16.2	11.6	4.7	9.7	2.7
Pakistan	yes	19.7	19.7	19.7	6.0	19.7	10.1	4.1
**Sub-Saharan Africa**								
Angola	no	18.3	18.3	18.3	4.5	18.3	8.5	2.9
Benin	no	16.0	16.0	7.7	6.4	16.0	2.6	1.8
Botswana	yes	27.4	27.4	27.4	4.8	3.4	12.5	1.5
Burkina Faso	yes	35.2	35.2	22.8	17.7	35.2	14.2	8.4
Burundi	yes	30.9	29.1	30.4	28.4	30.9	25.8	23.8
Cabo Verde	no	20.6	20.6	6.4	6.3	20.6	3.0	2.0
Cameroon	yes	7.4	7.4	7.3	7.3	2.4	3.6	1.7
Central African Republic	no	10.8	10.8	10.8	9.8	10.8	10.8	9.8
Chad	yes	33.1	33.1	33.1	29.6	33.1	31.5	28.1
Comoros	yes	28.2	28.2	28.2	5.3	28.2	1.8	1.2
Congo	no	14.3	14.3	2.6	2.0	14.3	3.2	1.2
Côte d'Ivoire	yes	28.3	28.3	6.3	6.3	4.8	6.2	1.4
Democratic Republic of the Congo	yes	38.8	38.8	38.8	20.0	33.2	35.4	16.1
Djibouti	no	3.2	2.4	2.9	2.1	3.2	1.4	1.2
Eswatini	yes	25.2	25.2	25.2	5.0	25.2	5.6	2.3
Ethiopia	yes	10.2	5.4	10.2	6.3	4.9	10.2	3.5
Gabon	no	13.0	13.0	13.0	2.4	5.5	3.0	1.1
Gambia	no	30.6	30.6	11.4	5.6	30.6	2.7	1.5
Ghana	no	12.4	12.4	5.6	5.6	12.4	3.3	2.2
Guinea	no	15.0	15.0	6.3	5.1	15.0	1.8	1.3
Guinea-Bissau	no	22.4	22.4	22.4	7.8	22.4	2.2	1.6
Kenya	yes	9.3	5.3	6.4	6.4	3.2	4.9	1.9
Lesotho	yes	34.2	34.2	12.5	8.4	3.6	24.3	2.0
Liberia	no	5.1	5.1	5.1	5.1	5.1	1.1	1.1
Madagascar	no	20.7	20.7	20.7	6.5	20.7	5.2	2.7

Country	Zinc deficiency as a public health problem	Current program[2], %	Full compliance[3], %	Aligned standards [4], %	New/ aligned standards with full compliance Wheat Flour only [5], %	New/ aligned standards with full compliance, Maize Flour only[5], %	New/ aligned standards with full compliance, Rice only[5], %	New/ aligned standards with full compliance, Combined[5], %
Malawi	yes	20.5	11.9	17.9	14.2	11.9	20.1	8.7
Mali	no	18.5	18.5	9.9	8.5	18.5	6.5	3.9
Mauritania	no	11.0	11.0	2.7	1.5	11.0	5.0	1.2
Mauritius	no	16.3	16.3	16.3	2.9	16.3	3.7	1.7
Mozambique	yes	15.4	11.1	5.0	4.1	7.7	4.0	1.5
Namibia	no	21.4	21.4	21.4	4.0	21.4	12.6	3.1
Niger	no	21.7	21.7	18.7	18.1	21.7	7.8	6.9
Nigeria	yes	9.1	7.5	5.7	4.3	9.1	2.9	1.9
Rwanda	yes	25.8	17.2	23.5	15.8	19.3	19.1	9.7
Sao Tome and Principe	no	30.1	30.1	30.1	3.0	30.1	3.3	1.3
Senegal	yes	24.4	24.4	7.9	7.7	24.4	2.0	1.4
Seychelles	no	13.1	13.1	13.1	3.2	13.1	13.1	3.2
Sierra Leone	no	22.6	22.6	22.6	8.1	21.9	3.7	2.3
South Africa	yes	7.2	6.3	2.0	3.1	2.5	4.2	1.4
Sudan	no	6.1	6.1	6.1	3.0	6.0	6.1	3.0
Togo	no	12.2	12.2	8.9	8.9	8.4	4.2	2.8
Uganda	no	11.3	7.8	7.6	7.9	4.4	9.3	3.1
United Republic of Tanzania	yes	16.7	10.7	9.4	8.9	4.8	16.7	3.4
Zambia	yes	36.9	36.9	36.9	20.8	6.4	33.9	4.5
Zimbabwe	yes	33.1	8.7	22.7	11.1	6.6	10.9	2.3
**Central Asia, North Africa and Middle East**								
Algeria	no	12.3	12.3	12.3	1.8	12.3	10.3	1.7
Armenia	no	12.1	12.1	12.1	2.7	12.1	10.8	2.6
Azerbaijan	no	17.6	17.6	17.6	3.0	13.2	16.2	2.6
Egypt	no	9.5	9.5	9.5	2.5	9.5	3.1	1.5
Georgia	no	24.3	24.3	24.3	3.3	10.4	21.8	2.4
Iran (Islamic Republic of)	yes	23.3	23.3	3.8	2.6	23.3	13.3	2.3
Iraq	no	20.2	20.2	20.2	1.8	20.2	6.7	1.3
Jordan	no	7.3	6.7	2.0	1.8	6.9	4.0	1.4
Kazakhstan	no	2.6	1.9	2.3	1.5	2.6	2.1	1.3
Kuwait	no	15.1	15.1	15.1	3.1	15.1	4.7	2.0
Kyrgyzstan	no	6.4	6.4	2.5	2.0	2.6	5.1	1.2
Lebanon	no	21.8	21.8	21.8	2.6	21.8	12.9	2.2
Libyan Arab Jamahiriya	no	19.2	19.2	19.2	2.1	19.2	19.2	2.1

Country	Zinc deficiency as a public health problem	Current program[2], %	Full compliance[3], %	Aligned standards [4], %	New/ aligned standards with full compliance Wheat Flour only [5], %	New/ aligned standards with full compliance, Maize Flour only[5], %	New/ aligned standards with full compliance, Rice only[5], %	New/ aligned standards with full compliance, Combined[5], %
Mongolia	no	1.8	1.6	1.4	1.2	1.8	1.8	1.2
Morocco	no	16.5	16.5	3.5	2.5	16.5	15.5	2.4
Oman	no	17.6	17.6	4.2	4.2	17.6	4.8	2.4
Saudi Arabia	no	25.7	25.7	25.7	3.5	9.7	7.9	1.9
Syrian Arab Republic	no	22.8	22.8	22.8	2.8	22.8	22.8	2.8
Tajikistan	no	14.9	14.9	14.9	1.6	6.6	6.3	1.1
Tunisia	no	14.7	14.7	14.7	2.0	14.7	13.7	1.9
Turkey	no	17.7	17.7	17.7	2.6	17.7	12.6	2.3
Turkmenistan	no	3.6	3.6	1.1	1.0	3.6	2.2	0.9
United Arab Emirates	no	22.4	22.4	22.4	4.5	21.6	8.2	3.1
Uzbekistan	no	4.3	1.7	4.3	1.4	4.3	3.5	1.3
Yemen	yes	25.9	25.9	2.5	1.7	25.9	9.1	1.3
**Central and Andean Latin America and Caribbean**								
Antigua and Barbuda	no	8.1	8.1	2.3	2.3	8.1	3.9	1.7
Bahamas	no	6.5	6.5	2.9	2.9	6.5	2.8	1.8
Barbados	no	10.2	10.2	3.3	3.3	10.2	4.5	2.2
Belize	no	16.2	16.2	3.8	3.8	16.2	4.0	2.0
Bolivia (Plurinational State of)	no	7.9	7.9	2.8	2.8	7.9	6.9	2.6
Colombia	yes	9.3	9.3	4.3	3.7	9.3	2.8	1.8
Costa Rica	yes	11.1	11.1	1.6	4.4	5.2	2.9	1.6
Cuba	no	12.9	12.9	4.6	4.6	12.9	4.1	2.4
Dominican Republic	no	12.0	12.0	3.2	3.2	12.0	12.0	3.2
Ecuador	yes	11.5	11.5	2.2	2.2	11.5	11.5	2.2
El Salvador	no	29.7	29.7	10.3	10.3	29.7	16.6	7.0
Grenada	no	9.3	9.3	2.8	2.8	9.3	4.9	2.1
Guatemala	yes	28.5	28.5	9.2	9.2	28.5	19.9	7.3
Guyana	no	9.5	9.5	2.9	2.9	9.5	9.5	2.9
Haiti	yes	29.1	29.1	6.8	4.9	23.0	3.1	1.6
Honduras	no	31.6	31.6	10.1	10.1	31.6	12.1	5.4
Jamaica	no	14.2	14.2	3.0	3.0	14.2	3.2	1.6
Mexico	yes	14.5	5.3	14.2	5.7	4.4	11.0	2.5
Nicaragua	no	26.7	25.7	7.1	9.6	26.7	15.4	6.7
Panama	no	5.0	5.0	1.6	2.8	5.0	2.3	1.6
Peru	no	16.9	11.8	5.3	5.0	16.9	7.6	3.2

Country	Zinc deficiency as a public health problem	Current program[2], %	Full compliance[3], %	Aligned standards [4], %	New/ aligned standards with full compliance Wheat Flour only [5], %	New/ aligned standards with full compliance, Maize Flour only[5], %	New/ aligned standards with full compliance, Rice only[5], %	New/ aligned standards with full compliance, Combined[5], %
Saint Lucia	no	7.1	7.1	2.2	2.2	7.1	4.7	1.9
Saint Vincent and the Grenadines	no	8.4	8.4	2.8	2.8	8.4	8.4	2.8
Suriname	no	16.8	16.8	2.8	2.8	16.8	16.8	2.8
Trinidad and Tobago	no	13.2	13.2	2.8	2.8	13.2	5.8	2.1
Venezuela (Bolivarian Republic of)	no	23.4	23.4	3.0	3.0	23.4	4.7	1.7
**East and Southeast Asia and Pacific**								
Cambodia	yes	19.2	19.2	19.2	15.2	18.7	19.2	14.8
Democratic People's Republic of Korea	no	41.8	41.8	41.8	12.0	41.8	3.2	2.3
Fiji	no	4.0	4.0	3.1	3.1	4.0	1.5	1.4
French Polynesia	no	7.1	7.1	7.1	2.8	7.1	2.5	1.6
Indonesia	yes	14.1	13.0	9.2	8.1	14.1	10.3	6.4
Kiribati	no	11.5	9.8	6.9	5.5	11.5	2.1	1.6
Lao People's Democratic Republic	no	11.1	11.1	11.1	8.8	11.1	5.8	5.0
Malaysia	no	12.7	12.7	12.7	3.7	12.7	4.7	2.2
Maldives	yes	25.9	25.9	25.9	4.1	25.9	4.7	2.1
Myanmar	no	10.4	10.4	10.4	6.9	10.4	9.7	6.5
New Caledonia	no	7.1	7.1	7.1	2.0	7.1	3.0	1.5
Papua New Guinea	no	13.4	13.4	4.7	3.5	13.4	3.7	1.8
Philippines	yes	11.2	11.2	2.3	2.9	11.2	3.9	1.8
Samoa	no	3.7	3.2	3.1	2.6	3.7	2.6	2.0
Solomon Islands	no	2.7	2.6	1.5	1.6	2.7	2.0	1.3
Sri Lanka	no	35.2	35.2	35.2	10.0	35.2	8.1	4.0
Thailand	no	17.7	17.7	17.7	6.8	17.7	2.8	2.1
Timor-Leste	yes	21.5	21.5	21.5	4.9	21.5	2.3	1.4
Vanuatu	no	14.1	14.1	14.1	3.9	14.1	11.0	3.4
Viet Nam	yes	5.7	5.0	5.8	5.1	5.7	3.7	3.5
**Central and Eastern Europe**								
Albania	no	5.7	5.7	5.7	2.1	5.3	4.3	1.8
Belarus	no	7.3	7.3	7.3	4.1	7.1	6.4	3.7
Bosnia and Herzegovina	no	11.8	11.8	11.8	4.0	4.3	10.7	2.3

Country	Zinc deficiency as a public health problem	Current program[2], %	Full compliance[3], %	Aligned standards [4], %	New/ aligned standards with full compliance Wheat Flour only [5], %	New/ aligned standards with full compliance, Maize Flour only[5], %	New/ aligned standards with full compliance, Rice only[5], %	New/ aligned standards with full compliance, Combined[5], %
Bulgaria	no	13.0	13.0	13.0	2.6	3.8	10.6	1.6
Croatia	no	7.3	7.3	7.3	2.8	5.4	6.5	2.3
Czechia	no	8.8	8.8	8.8	2.7	8.8	6.9	2.4
Estonia	no	8.0	8.0	8.0	3.7	5.7	7.2	2.9
Hungary	no	6.9	6.9	6.9	1.9	6.9	5.5	1.8
Latvia	no	9.7	9.7	9.7	3.6	9.4	8.5	3.3
Lithuania	no	6.7	6.7	6.7	2.0	6.4	5.8	1.9
Montenegro	no	4.9	4.9	4.9	2.0	4.5	4.6	1.8
North Macedonia	no	13.5	13.5	13.5	3.4	4.7	11.6	2.1
Poland	no	8.5	8.5	8.5	2.5	8.5	7.8	2.4
Republic of Moldova	no	17.7	17.7	17.4	4.0	3.3	14.6	1.8
Romania	no	7.8	7.8	7.8	2.7	3.5	6.7	1.8
Russian Federation	no	6.1	6.1	6.1	1.5	6.0	4.8	1.4
Serbia	no	8.5	8.5	8.5	3.3	3.2	8.0	1.9
Slovakia	no	14.0	14.0	14.0	2.7	14.0	11.7	2.5
Slovenia	no	13.5	13.5	13.5	3.5	4.6	11.3	2.1
Ukraine	no	11.1	11.1	11.1	2.5	11.1	9.8	2.4
**China**								
China	no	6.6	6.6	6.6	3.2	6.6	2.4	1.7
**Southern and Tropical Latin America**								
Argentina	no	2.0	2.0	1.0	1.0	1.6	1.6	0.9
Brazil	no	6.2	6.2	2.4	2.6	2.6	2.5	1.2
Chile	no	5.5	5.5	1.7	1.7	5.5	3.7	1.5
Paraguay	no	15.5	15.5	3.6	3.6	15.5	9.8	2.9
Uruguay	no	5.7	5.7	3.6	1.8	5.7	4.5	1.6
**High-income**								
Australia	no	4.6	4.6	2.3	2.1	3.9	3.3	1.6
Austria	no	8.2	8.2	8.2	2.8	5.6	6.8	2.3
Belgium	no	7.4	7.4	7.4	2.1	7.1	4.4	1.7
Canada	no	9.3	9.3	3.5	3.3	9.3	6.0	2.7
Cyprus	no	10.1	10.1	10.1	2.5	6.0	7.8	1.9
Denmark	no	6.4	6.4	6.4	2.9	6.4	5.4	2.6
Finland	no	5.1	5.1	5.1	2.5	5.1	4.4	2.3
France	no	5.1	5.1	5.1	1.8	5.1	4.1	1.6

Country	Zinc deficiency as a public health problem	Current program[2], %	Full compliance[3], %	Aligned standards [4], %	New/ aligned standards with full compliance Wheat Flour only [5], %	New/ aligned standards with full compliance, Maize Flour only[5], %	New/ aligned standards with full compliance, Rice only[5], %	New/ aligned standards with full compliance, Combined[5], %
Germany	no	9.3	9.3	9.3	3.1	9.3	7.8	2.9
Greece	no	8.6	8.6	8.6	2.5	8.6	7.4	2.3
Iceland	no	2.8	2.8	2.8	1.6	2.8	2.6	1.5
Ireland	no	4.6	4.6	4.6	1.7	3.4	4.0	1.5
Israel	no	4.9	4.9	4.9	1.7	4.9	2.8	1.4
Italy	no	7.3	7.3	7.3	2.3	7.3	5.6	2.1
Japan	no	17.6	17.6	17.6	5.3	17.6	3.6	2.2
Luxembourg	no	5.6	5.6	5.6	2.5	5.3	4.9	2.3
Malta	no	8.0	8.0	8.0	2.8	6.8	6.6	2.4
Netherlands	no	6.2	6.2	6.2	2.6	6.2	5.2	2.4
New Zealand	no	7.1	7.1	4.2	2.4	5.8	4.5	1.8
Norway	no	6.5	6.5	6.5	2.4	6.5	5.4	2.2
Portugal	no	6.6	6.6	6.6	2.7	6.6	4.0	2.1
Republic of Korea	no	10.0	10.0	10.0	4.1	10.0	10.0	4.1
Spain	no	6.7	6.7	6.7	2.4	6.7	4.5	2.0
Sweden	no	6.3	6.3	6.3	2.2	6.3	4.8	2.0
Switzerland	no	5.4	5.4	5.4	2.1	5.4	4.9	2.0
United Kingdom of Great Britain and Northern Ireland	no	5.9	5.9	2.2	2.1	5.1	4.7	1.8
United States of America	no	5.8	5.8	1.9	2.4	4.0	4.2	1.8

[1]Estimates were calculated using the composite nutrient composition database, IZiNCG physiological requirements, the Miller Equation to estimate zinc absorption[38] and an assumed 25% inter-individual variation in zinc intake[22]. Although not a primary objective of this analysis, estimates from each hypothetical scenario are presented for all countries in this supplementary table, to make information available to relevant stakeholders.

[2]Current program scenario reflects fortification as currently implemented in all countries with current mandatory or voluntary fortification standards (whether or not zinc fortification is included in the standards) for wheat flour, maize flour and/or rice (current zinc standard, current % compliance) (n = 87). For all other countries (n = 87), results from the "current program" scenario are equivalent to the "baseline" scenario.

[3]Full compliance scenario reflects retaining current standards (whether or not zinc fortification is included in the standards), but compliance with mandatory fortification is increased to 100%. Scenario applied only to countries with current mandatory fortification standards. Scenario applied to all countries, whether or not they were identified as having zinc deficiency as a public health problem (n = 87); in actuality, estimated prevalence of inadequate zinc intake only changed from the "current program" scenario for countries with zinc fortification included in the standards. For all other countries (n = 87), results from the "current program" were retained.

[4]Aligned standards scenario reflects either adding zinc to the mandatory standard (if it is not already included), and aligning the standard to reflect current zinc fortification guidelines or recommendations. Scenario applied only to countries with current mandatory fortification standards. Scenario applied to all countries, whether or not they were identified as having zinc deficiency as a public health problem (n = 87). For all other countries (n = 87), “current program” were retained.

[5]New/aligned standards with full compliance scenario reflects mandatory zinc fortification standards, aligned with current guidelines or recommendations, for each staple food independently and combined, and having 100% compliance with the standards. Scenario applied to all countries, whether or not they were identified as having zinc deficiency as a public health problem (n = 174)
